# Reverse-Phase Microarray Analysis Reveals Novel Targets in Lymph Nodes of *Bacillus anthracis* Spore-Challenged Mice

**DOI:** 10.1371/journal.pone.0129860

**Published:** 2015-06-19

**Authors:** Taissia G. Popova, Virginia Espina, Lance A. Liotta, Serguei G. Popov

**Affiliations:** 1 Center for Applied Proteomics and Molecular Medicine, George Mason University, Manassas, Virginia, United States of America; 2 National Center for Biodefense and Infectious Diseases, George Mason University, Manassas, Virginia, United States of America; University of Pittsburgh, UNITED STATES

## Abstract

Anthrax is a frequently fatal infection of many animal species and men. The causative agent *Bacillus anthracis* propagates through the lymphatic system of the infected host; however, the specific interactions of the host and microbe within the lymphatics are incompletely understood. We report the first description of the phosphoprotein signaling in the lymph nodes of DBA/2 mice using a novel technique combining the reverse-phase microarray with the laser capture microdissesction. Mice were challenged into foot pads with spores of toxinogenic, unencapsulated Sterne strain. The spores quickly migrated to the regional popliteal lymph nodes and spread to the bloodstream as early as 3 h post challenge. All mice died before 72 h post challenge from the systemic disease accompanied by a widespread LN tissue damage by bacteria, including the hemorrhagic necrotizing lymphadenitis, infiltration of CD11b+ and CD3+ cells, and massive proliferation of bacteria in lymph nodes. A macrophage scavenger receptor CD68/macrosialin was upregulated and found in association with vegetative bacteria likely as a marker of their prior interaction with macrophages. The major signaling findings among the 65 tested proteins included the reduced MAPK signaling, upregulation of STAT transcriptional factors, and altered abundance of a number of pro- and anti-apoptotic proteins with signaling properties opposing each other. Downregulation of ERK1/2 was associated with the response of CD11b+ macrophages/dendritic cells, while upregulation of the pro-apoptotic Puma indicated a targeting of CD3+ T-cells. A robust upregulation of the anti-apoptotic survivin was unexpected because generally it is not observed in adult tissues. Taken together with the activation of STATs it may reflect a new pathogenic mechanism aimed to delay the onset of apoptosis. Our data emphasize a notion that the net biological outcome of disease is determined by a cumulative impact of factors representing the microbial insult and the protective capacity of the host.

## Introduction

Anthrax is a lethal disease of many animal species and men. It is caused by the Gram-positive bacterium *Bacillus anthracis* first discovered by Davaine and Rayer in 1850. The microbe is endemic to different geographic regions all over the world and can be found in soil in the form of the infectious spores highly resistant to environmental conditions [1]. The exposure to the spores can occur through the cutaneous abrasions or wounds, inhalation, and alimentary route. Due to the effective veterinary and public- health measures the incidence of human cutaneous and inhalation anthrax in developed countries is low. However, anthrax remains a major concern because of the potential intentional release of biological weapons containing *B*. *anthracis* spores [1].

Historically, anthrax research has been focused mainly on the inhalation form due to its high mortality. It is likely that patients unaware of the exposure to infectious aerosol would start seeking help only after the initial prodromal period, when the initial flu-like symptoms aggravate due to the onset of the systemic stage of disease. At this stage, even the most advanced antibiotic therapies provide only about 50% survival [1,2]. Although the potency of the spores to establish infection is substantially different between the entry routes, humans and experimental animals almost invariably demonstrate the onset of a generalized disease before death associated with the toxemia and the presence of bacilli in blood and other organs. Independently of the spore entry route, *B*. *anthracis* first spreads *via* lymphatics before appearing in the bloodstream. Recent studies have supported these observations using the light-producing *B*. *anthracis* which allowed direct detection using sensitive camera of bacteria in regional draining lymph nodes before their appearance in the bloodstream [3,4].

Although it has been firmly established that the lymphatic system can serves as the conduit by which germinating *B*. *anthracis* spores are delivered to the sentinel LNs within hours after inhalation exposure, there is disagreement regarding the specific mechanisms involved [4]. The Trojan horse model of infection assumes that upon inhalation exposure to the aerosolized spores the alveolar macrophages (MΦs) play role in transporting the engulfed spores to the LNs with little or no damage to the lung. On the other hand, the jailbreak model suggests that the intracellular transport of spores to the draining LNs is not required. Instead, it assumes that *B*. *anthracis* spores first germinate and multiply at favorable sites of entry into the host (such as an airway lumen, nasal or gut-associated lymphoid tissues). During this process, virulence factors produced by bacteria dampen the immune response and damage tissues, allowing bacterial dissemination into the regional LN. While both models recognize the important role of LNs which can act as holding reservoirs from where the proliferating bacteria can spread to other organs of the body, the specific protein environment of LN making it a niche for survival and fast bacterial multiplication has not been characterized. There is a paucity of data regarding which bacterial pathogenic factors beyond the lethal toxin (LeTx) and edema toxin (EdTx) considered to be the major culprits can promote survival of bacteria during anthrax lymphadenitis and which host mediators are involved in hindering it. From a clinical prospective, the Trojan horse model predicts that interaction of bacteria with the immune system in the LNs represents a critical point at which intervention could prevent transition to a lethal systemic disease. However, no specific treatment or prophylaxis of anthrax lymphadenitis is available to support this conclusion. It has to be emphasized that commonly administered antibiotics poorly penetrate into LNs. Viable bacilli were found in patient's LNs after death, while the blood stream was sterile after antibiotic therapy [5]. Therefore, better understanding of how the disease becomes initiated and disseminated through the lymphatics is necessary for the improvement of medical protection and treatment means against anthrax [2].

To address these knowledge gaps we proposed to reveal aberrations caused by anthrax infection in the LNs using a cutaneous infection murine model in which the spores can be quickly delivered to the popliteal LNs by a foot pad injection for the assessment of subsequent host responses. Previously, we used this model to carry out the whole proteome analysis of the intra-nodal lymph from the infected mice and revealed a large number of proteins induced by the disease. Among those we detected the presence of soluble phosphoproteins participating in the intracellular signaling pathways and suggested that they may reflect the host cell damage by the pathogenic factors of *B*. *anthracis*. The phosphoprotein signaling plays central role in cellular biology and host response to infection. Therefore, we anticipated that elucidation of the phosphorylation-driven signaling pathways in LN tissues during anthrax infection would provide important information about the disease. For this purpose we used the reverse-phase protein microarray (RPMA) combined with the laser capture microdissection (LCM) previously developed in our laboratory for multiplexed analysis of phosphorylation-driven cell signaling cascades in tissues of cancer patients [6–8].

RPMA is a novel protein microarray approach that can quantify proteins and post-translationally modified proteins with femtomolar sensitivity using specific antibodies, from nanoliter volumes of samples printed on the slides by a robotic device [6,7]. Our current and continuously expanding repertoire includes more than 300 validated antibodies to proteins and post-translationally modified epitopes relevant to multiple signaling pathways including apoptosis, inflammation, and immune response. The method is able to generate large amount of data in a very short time frame when compared to conventional immunological methods such as Western blots and ELISAs. The RPMA technique is highly sensitive and therefore allows investigation of the *in situ* microenvironment in specific anatomical locations such as draining LNs using small amounts of tissue harvested by LCM.

The LCM is a technique for dissecting heterogeneous tissue sections, cytological preparations, or even live cells for isolation of tissue fragments or cell populations *via* direct visualization of the cells [8]. The instrument generates laser pulses to activate a thermoplastic polymer film that expands and surrounds the cells of interest. This polymer-cell composite can be lifted from the slide, effectively microdissecting the cells of interest. In the past, molecular signaling studies of the murine LN tissues met a number of experimental complications relevant to a small sample size and low sensitivity of the analytical methods, restricting access to the *in vivo* proteome of the infected tissue. The combination of RPMA and LCM allowed us to overcome these barriers and provided means to directly study the molecular fluctuations within the *in situ* microenvironment that incorporate the full complexity of bacteria-host interactions.

In this report we described the progression of anthrax infection in the regional popliteal LNs and characterized the phosphoproteomic signaling by 65 host proteins reflecting interaction of the lymphatic system with *B*. *anthracis*. In order to correlate the responses with the histopathological features of infection, we immunostained the LN tissue sections and obtained additional information regarding the cell types involved in the observed signaling.

## Materials and Methods

### Reagents and antibodies

The rabbit antibodies against total and phosphorylated forms of the following human cross-reacting with corresponding mouse proteins used for reverse phase protein microarrays were from Cell Signaling Technology (Beverly, MA) unless otherwise noted and used at the dilutions indicated: 1:50 for STAT5 (Tyr694), Caspase-7 cleaved (Asp198), p38 MAP Kinase (Thr180/Tyr182), Caspase-3 cleaved (Asp175), e-NOS (Ser113), e-NOS (Ser1177), NF-kappaB p65 (Ser536), p70 S6 kinase (Ser371), p70 S6 kinase (Thr389), Ship1 (Tyr1020); 1:100 for Bad, Bim, CD68, Chk2 (Ser33/Ser35), LC3B, Lck (Tyr505) (Life Technologies, NY, USA), myeloperoxidase (Abcam, MA, USA), HSP27 (Ser82), STAT1, STAT3 (Tyr705), STAT3 (Ser727), CREB (Ser133), AKT, AKT (Ser473), Bad, p53 (Ser15), Caspase-9 cleaved (Asp330), STAT6 (Tyr641), XIAP, Puma, FLIP; 1:200 for ERK1/2, p38 MAP kinase, PTEN (Ser380), STAT5 (Tyr694), PARP cleaved (D214), ERK1/2 (Thr202/Tyr204), Beclin 1, FADD (Ser194), Sumo1, S6 Ribosomal protein (Ser235/Ser236), STAT6; 1:250 for Src (Tyr416), Src (Tyr527), GSK-3α/β (Ser21/9); 1:500 for SAPK/JNK, PTEN, Bcl-xL, Survivin, SAPK/JNK (Thr183/Tyr185), HSP90, ATF2 (Thr69/Thr71), pIKBα; 1:1000 for Caspase 8 (EMD Millipore, Billerica, MA, USA), TLR9, STAT1 (Tyr701), STAT3, Bax, Bim, S6 Ribosomal protein (Ser240/Ser244), Ship1 (Tyr1020); 1:2000 for IL-10 (Abcam, MA, USA), Sumo2/3, Proteosome 20S. Other reagents were from Sigma-Aldrich (St Louis, MO).

### Synthesis of tracer fluorescent nanoparticles

The nanoparticles (NPs) we synthesized are based on N-isopropylacrylamide (pNIPAm) and methylenebisacrylamide (BIS) as a cross-linker co-polymerized with allylamine (AA) for incorporation of fluorescent label. The synthesis was carried out *via* precipitation polymerization essentially as described [9–11]. NIPAm (9.0 g) and BIS (0.28 g) were dissolved in 250 ml of water, and the solution was then partially degassed by vacuum filtration through a 0.45 μm nylon filter. The filtered solution was purged with nitrogen at room temperature and a medium rate of stirring for 15 min, before AA (670 μl, 12 μmoles) was added to the reaction. Following the addition of AA, the solution was purged with nitrogen for another 15 min and then heated to 75°C. Once the reaction mixture had attained a stable temperature of 75°C, polymerization was initiated with the addition of potassium persulfate (0.1 g) in 1.0 ml of water. The reaction was maintained at a constant temperature of 75°C with stirring under nitrogen for 3 h. After this time, the reaction was allowed to cool to room temperature overnight with stirring under nitrogen. The particles were then harvested and washed by centrifugation for 20 min at 23°C and 16,000 g with the supernatant subsequently discarded. The pelleted particles were then re-suspended in 300 ml of water, and the suspended particles pelleted by centrifugation. This centrifugation-redispersion process was repeated for a total of 5 times. Particles were stored as a suspension in water with a few drops of chloroform as an antimicrobial. The succinimidyl ester of Alexa Fluor 555 was used for conjugating the dye to primary amines on pNIPAm-co-AA NPs. For this purpose 100 μl of NPs were washed 2x1 ml of 50 mM bicarbonate buffer, pH 8.3, re-suspended in 500 μl of the buffer, and mixed with 50 μl of the dye solution (1 mg in 100 μl of DMF). After 1 h at room temperature the particles were washed 3x1 ml of PBS (pH 7.4) and finally re-suspended in 500 μl of PBS. The particles were observed at 555/570 nm using Olympus BX51 microscope with a TRITC filter set. The number of labeled NPs was counted after appropriate dilution and was found to be about 7x10^5^ per 1 μl of original suspension. For injection into the hind leg footpads of mice the NP suspension was mixed with equal volume of 2% tracer dye Evans Blue in PBS. This dye allowed location of the LNs during surgery and did not quench the fluorescence of Alexa Fluor 555.

### Animal challenge and extraction of proteins from LNs

All animal procedures were approved by the George Mason University Institutional Animal Care and Use Committee. All surgery was performed after carbon dioxide asphyxiation, and all efforts were made to minimize suffering. Female 6- to 8-week-old DBA/2J mice (Jackson Labs) received food and water *ad libitum* and were challenged with *B*. *anthracis* Sterne 34F2 or delta-Sterne (dSterne) spores (4x10^6^ spores in 20 μL of PBS, intradermally into both hind footpads, 3 animals per challenge group) on day 0. The spores were prepared as described [12]. The Sterne strain is fully toxinogenic but strongly attenuated due to the lack of a polypeptide capsule. The dSterne strain is an isogenic derivative of 34F2 strain. It contains no plasmid and is non-virulent. The Sterne strain was obtained from Colorado Serum Co., and the dSterne strain from the collection of the George Mason University National Center for Biodefense and infectious Diseases. Survival of animals was monitored for 4 days. Thirty min before euthanasia the animals were anesthetized with isoflurane and 20 μl of a mixture containing 1% tracer dye Evans Blue in PBS were injected into foot pads. In some experiments the dye solution contained the Alexa Fluor 555-labelled NPs. The LNs were surgically removed into 10% neutral buffered formalin solution for a histological evaluation and LCM. Control non-infected animals received equal volume of PBS. To determine a bacterial load the spleens and LNs were homogenized on ice using frosted glass slides. The tissues were re-suspended in ice-cold PBS and plated in different dilutions onto LB agar plates. The plates were incubated at 37°C overnight. The number of colonies grown reflected the number of plaque-forming units (PFUs) representing viable spores and vegetative bacterial cells. To determine the number of heat-resistant ungerminated spores the tissue homogenates were transferred from ice to 65°C and incubated for 30 min before plating.

### Immunohistochemical analysis

After fixing in formalin, the tissues were embedded in paraffin, the paraffin blocks were sliced into 5 μm sections, and mounted onto glass slides for standard hematoxylin/eosine (H&E) staining and further immunohistochemical evaluation. The slides were subjected to the procedure of antigen retrieval by incubating them for 20 min in citrate buffer (15 mM citric acid, pH 6.0) at 95°C. Sections after antigen retrieval were incubated in 3% hydrogen peroxide in methanol for 5 min to inhibit peroxidase activity, blocked with Dako Protein Block (Dako) for 5 min, and then incubated with an appropriate primary antibody for 30 min, followed by Dako anti-rabbit EnVision+ HRP-Labeled Polymer (Dako). All precedures were carried out using an automatic slide stainer (Autostainer, Dako Cytomation, Carpinteria, CA) and the manufacturer-supplied reagents. Colorimetric detection was completed with diaminobenzidine for 7 min, and slides were counterstained with hematoxylin.

To analyze the presence of bacteria within the LNs, the slides were then stained with rabbit anti*-B*. *anthracis* immune serum (dilution 1:100). The anti-*B*.*anthracis* serum was obtained from rabbits immunized with spores of the Sterne strain and was shown by us to recognize a vegetative form of the bacterium.

### LCM and RPMA analysis

To prepare samples for RPMA analysis the paraffin blocks of formalin-fixed LNs were cut into 5 μm slices, mounted onto the polyethylene naphthalate (PEN) membrane slides, stained with hematoxylin and used the Arcturus XT LCM system with CapSure Macro Caps (Applied Biosystems) to cut out pieces of tissue corresponding to the whole content of LNs below the capsule layer. The cutout material from LCM Caps was lysed in the protein lysis buffer: 4.5 volumes of T-PER Tissue Protein extraction reagent (Pierce) and Novex Tris-glycine 2X SDS loading buffer (Invitrogen), and 1 volume of TCEP (Tris-2-carboxyethylphosphine) Bond Breaker, 10% v/v (Pierce). The Arcturus XT-system combines IR capture microdissection and UV laser cutting in one instrument, which allows the “cut and capture” of cells of interest by first ablating unwanted cells, thus preventing contamination during cell capturing. The cutout material from 3 LNs (30 slices from each LN) was combined and finally boiled for 10 min before printing onto nitrocellulose RPMA slides (Whatman, MA). Three nl of each sample were arrayed by direct contact printing using a high-resolution 2470 arrayer (Aushon Biosystems, Billerica, MA). Samples were printed as duplicates of the four-point serial dilution curves to ensure a linear detection range for the antibody concentrations used. Slides were stored with desiccant (Drierite, W. A. Hammond, Xenia, OH, USA) at -20°C before analysis with antibodies. To estimate the total protein amount, selected slides were stained with Sypro Ruby Protein Blot Stain (Molecular Probes, Eugene, OR) and visualized on a Fluorchem imaging system (Alpha Innotech, San Leandro, CA) equipped with a Cy3 filter. Slides were stained with specific antibodies on an automated slide stainer (Dako, Carpinteria, CA) using a biotin-linked peroxidase-catalyzed signal amplification. The arrayed slides were placed into 1x Re-Blot solution (Chemicon, Temecula, CA) for 15 min, washed two times for 5 min each in PBS, placed into I-Block solution (Applied Biosystems, Foster City, CA) in PBS/0.1% Tween-20 for at least 2 h, and then immunostained using an automatic slide stainer (Autostainer, Dako Cytomation, Carpinteria, CA) and the manufacturer-supplied reagents. Briefly, the slides were incubated for 5 min with hydrogen peroxide, rinsed with high-salt Tris-buffered saline (CSA Buffer, Dako) supplemented with 0.1% Tween-20, blocked with avidin block solution for 10 min, rinsed with CSA buffer, and then incubated with biotin block solution for 10 min. After another CSA buffer rinse, 5 min incubation with Protein Block solution was followed by air-drying. The slides were then incubated with either a specific primary antibody diluted in Dako Antibody Diluent or, as a control, with only Dako Antibody Diluent for 30 min. Prior to using in the RPMA, every antibody underwent extensive validation for specificity [13]. The slides were then washed with CSA buffer and incubated with a secondary biotinylated goat anti-rabbit IgG H+L antibody (1:10,000) (Vector Labs, Burlingame, CA) for 15 min. For amplification purposes, the slides were washed with CSA buffer and incubated with streptavidin-horseradish peroxidase for 15 min, followed by a CSA buffer rinse. Slides were then incubated for 5 min in diaminobenzidine chromogen diluted in Dako DAB diluent, washed in deionized water and imaged using UMAX 2100XL flatbed scanner (UMAX, Dallas, TX) using the following settings: white balance 255, black 0, middle tone 1.37, 600 dpi, 14 bit.

Spot intensity was analyzed by Image Quant v5.2 software (Molecular Dynamics). Data reduction was performed with RPMA Analysis Suite (http://capmm.gmu.edu/rpma-analysis-suite). To normalize data, the relative intensity value for each endpoint for each spot was divided by the relative intensity value for the total cellular protein. The 95% confidence interval (CI) for each protein was calculated using the Student’s t-test for 3 independent samples at each time point. An average CI for all protein data as a characteristic of its variability between different proteins was 8.4 +/- 5.7% (*p* = 0.95). Based on this, changes in the protein levels above 14% were considered statistically reliable.

## Results

### Propagation of infection through lymphatics

Footpad inoculation provides a combination of intradermal and subcutaneous routes for administration of vaccines, drugs and infectious agents in the mouse models. The path of draining lymph from footpad is well characterized and allows direct delivery to three locations for analyses of lymph node responses: popliteal, inguinal, and sub-iliac [14].

In naïve mice the process of the lymph flow through the lymphatics in the absence of inflammatory responses, which may influence the passage of the lymph fluid through the channels in the sinuses [15], appears to be rather fast. Control experiments showed that detectable amounts of a tracer dye Evans Blue reached the popliteal and inguinal LNs in less than 30 min after injection. We also used the fluorescent hydrogel NPs of an average size of 600–700 nm to mimic the anthrax spores of similar size and detect the LN areas accessible to the spores. The fluorescence was found distributed in the subcapsular regions in agreement with the NP flow with the afferent lymph [16] (S1 Fig). However the majority of the NPs migrated further through the LN sinuses and became retained in the medullary and paracortex locations, consistent with their role in the particle filtration by the LNs [15,17].

**Fig 1:**
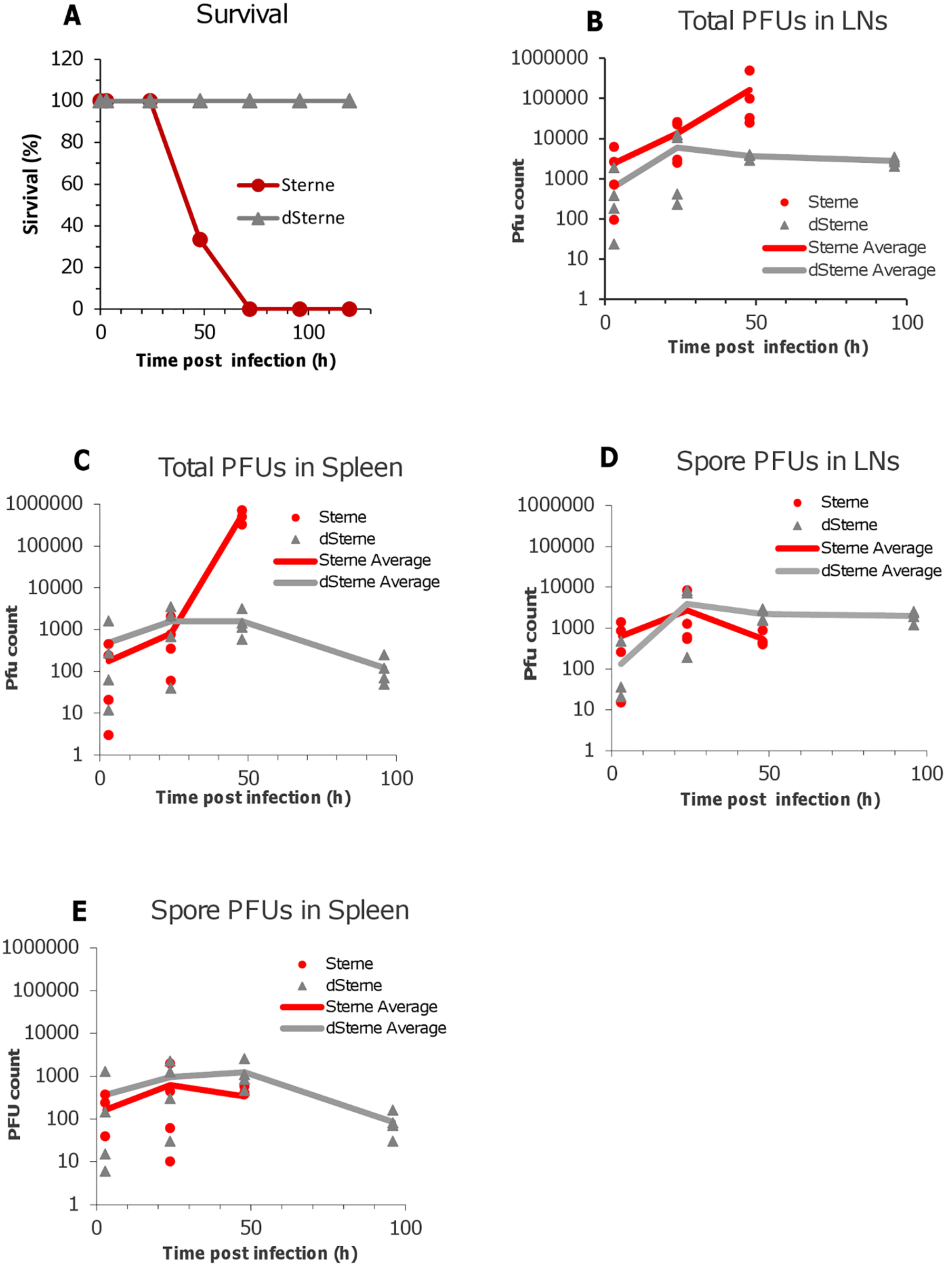
Mortality curves (A) and bacterial load in LNs and the spleen of spore-challenged DBA/2J mice (B-E). Animals were challenged with the toxinogenic, non-encapsulated *B*. *anthracis* Sterne 34F2 or non-toxinogenic, non-encapsulated delta-Sterne (dSterne) spores (4x10^6^ spores in 20 μL of PBS, intradermally into both hind footpads, 3 animals per challenge group. In (B) at the indicated times the animals were anesthetized with isoflurane and 20 μl of a mixture containing 1% tracer dye Evans Blue in PBS were injected into foot pads. The LNs were surgically removed and homogenized for the PFU determination on agar plates before and after heat inactivation of the vegetative bacteria.

In our challenge experiments a suspension of spores in 20 μl of PBS was injected into each of the hind footpads and the time course of dissemination of infectious material to draining popliteal LN and spleen was tested by seeding the organ homogenates onto Luria broth agar plates. Fig 1 shows the mortality curves and the results of the bacterial load analysis. At 3 h post challenge a large number of plaque-forming units (PFUs) representing viable spores and vegetative cells were detected in the LNs for both the virulent Sterne and non-virulent dSterne strains (Fig 1B). The amount of infectious material continued to increase steadily during infection with Sterne spores until all mice succumbed to the disease at 72 h (at this time point no survived animals could be tested). In the case of non-virulent dSterne strain the upward trend detectable during the initial phase of infection was replaced with a slow decline indicating gradual elimination of bacteria.

Remarkably, the infectious material was detected in the spleen in the amount and with the dynamics comparable to those in the LNs (Fig 1C and 1E). It demonstrated that the lymphatic system had insufficient filtering capacity allowing dissemination of infection to the bloodstream (however, in the amount of less than 0.002% of the initial footpad inoculum). The comparison of the bacterial load in the LN and the spleen clearly showed that while at 24 h post inoculation the infectious material was found predominantly in the LNs, the majority of proliferating bacteria at 48 h post infection were present in the spleen indicating the progression of disease to the systemic stage. The numbers of non-germinated, heat-resistant spores in the LNs continued to grow during the first day of infection (Fig 1D). It can be explained by an incomplete germination at the site of inoculation which served as a reservoir feeding the lymphatics with spores. A similar trend of lower magnitude was detected in the spleen (Fig 1E).

### Histopathological assessments of infected LNs

**Fig 2:**
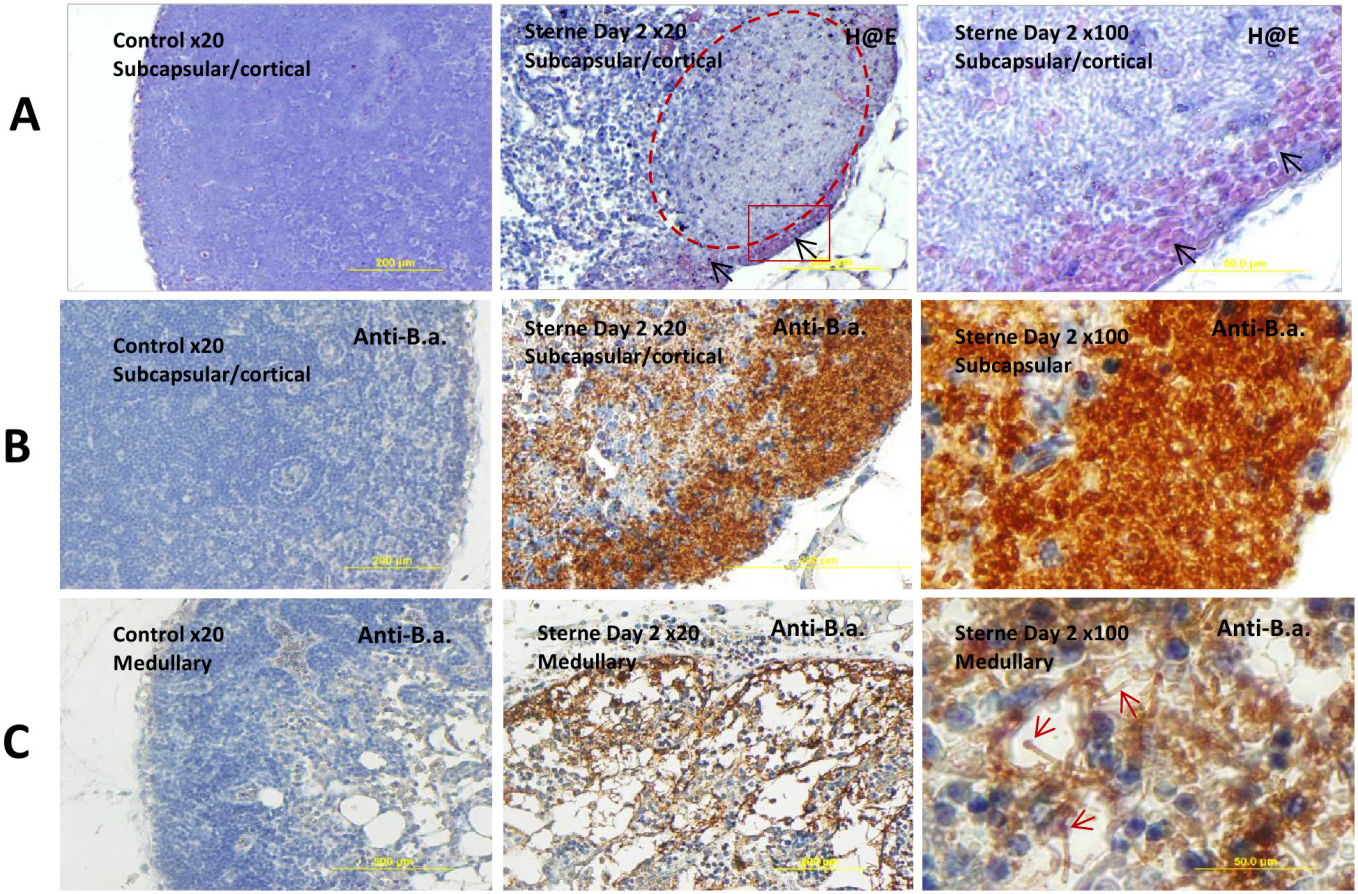
Staining with H&E (A) and anti-B. anthracis serum (B, C) of the popliteal LNs from control (left panels) and *B*. *anthracis*-challenged mice at day 2 post infection (middle and right panels). (A) Subcapsular hemorrhage (black arrows), massive amount of Sterne bacteria in a cortical region (dashed area in the middle panel), almost complete depletion of lymphocytes and necrosis in the sites of bacterial multiplication. Squared area in the middle panel is magnified in the right panel demonstrating numerous bacterial chains (gray/blue shadows) along with hemorrhage (black arrows). (B, C) Numerous bacteria (brown) are tightly packed in the subcapsular region and spread partially to the cortical zone (B). Medullary sinuses allow distinguish protruding bacterial chains (red arrows) with terminal “caps”(C).

Analysis of the H&E-stained LN tissue slides confirmed massive proliferation of the Sterne bacteria. At day 2 post challenge the bacteria were found spreading over large zones in LN parenchyma from the subcapsular and medullar regions (where they were initially delivered by the afferent lymph flow) as well as the cortical areas (Fig 2). The bacterial zones demonstrated a strong depletion of the hematoxylin nuclear staining characteristic of karyolysis and tissue necrosis. Intense tissue damage was further evidenced by large hemorrhages stained pink with eosin.

**Fig 3:**
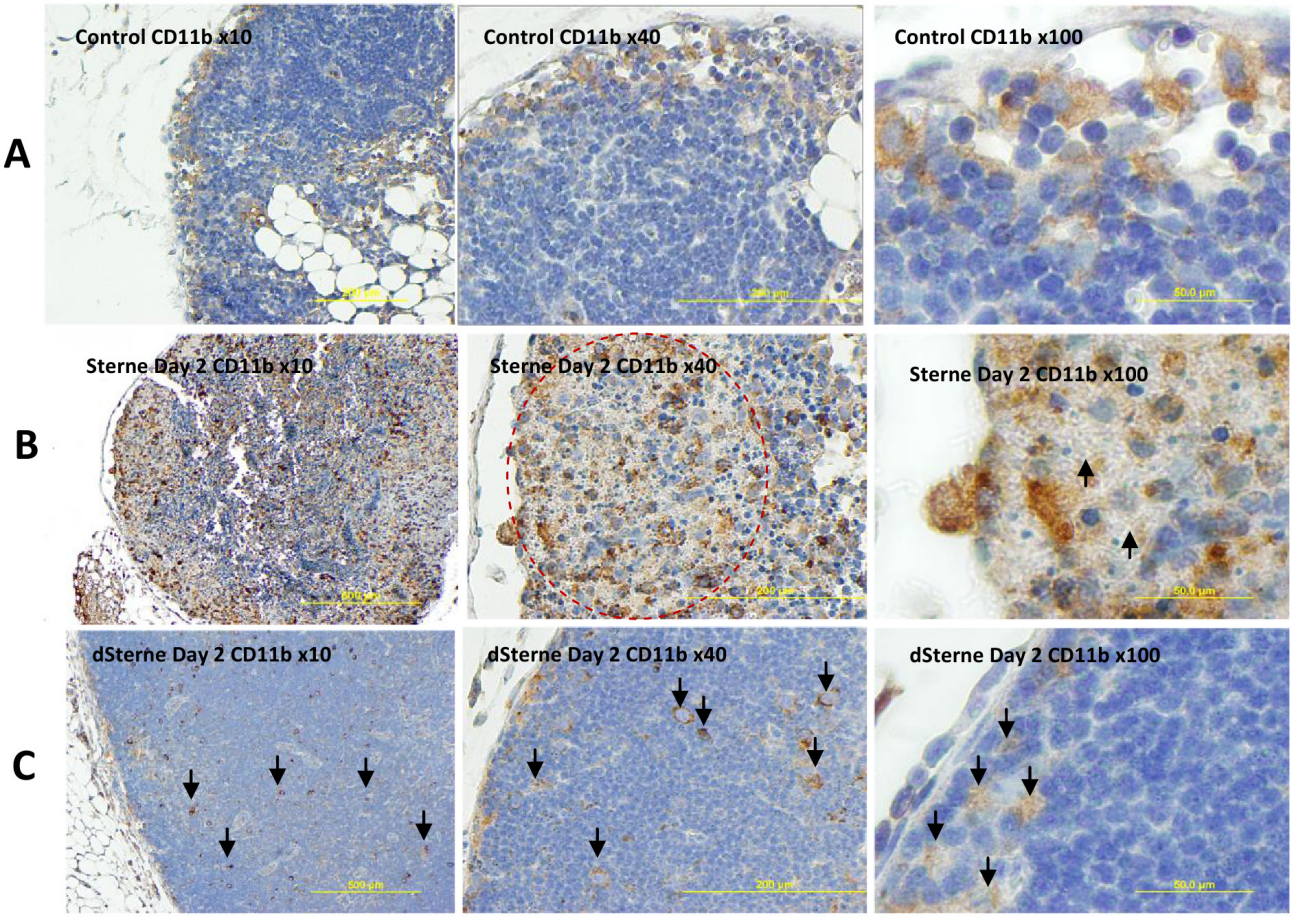
Immunohistochemical analysis of CD11b in formalin-fixed sections of popliteal LNs of naïve and *B*. *anthracis*-challenged mice. (A) In naïve mice the CD11b+ cells with monocyte/macrophage morphology were localized to subcapsular and medullar regions. (B) In the Sterne strain-infected mice at day 2 post infection the amount of CD11b antigen (stained brown) was increased and associated mainly with the areas of bacterial growth (circled in the middle panel). The LNs demonstrated a widespread cell death and tissue necrosis. Numerous bacterial chains are visible as gray shadows (arrows, right panel). (C) The LN tissue of the dSterne-infected mice demonstrated no gross pathology and the isolated CD11b+ cells scattered through LN parenchyma (arrows).

The bacterial proliferation was accompanied by massive infiltration of CD11b+ and CD3+ cells. The CD11b is a cell activation-dependent, pan-myeloid marker expressed mainly on monocytes, MΦs, and microglia. To a lower extent it can be found on granulocytes, NK cells, and subsets of dendritic cells (DCs). In our experiments the neutrophils stained negative, while the CD11b+ cells demonstrated the monocyte/ MΦ/DC morphology (S2 Fig). In the naïve mice the CD11b antigen was detected in the well-defined areas in the subcapsular and medullary regions (Fig 3A). In the Sterne-infected, pre-mortal mice at day 2 post challenge, the amount of CD11b antigen in LNs was increased all over the LN parenchyma including the cortical and paracortical areas (Fig 3B). Overall, the histopathological picture demonstrated a widespread tissue necrosis and massive destruction of the CD11b+ cells infiltrating in large numbers in response to the proliferating bacteria. It reflected inability of the host to control the virulent Sterne infection. In contrast, the non-virulent dSterne bacteria induced appearance of CD11b+ cells which was not accompanied by detectable tissue damage (Fig 3C). The bacterial cells identified in the tissue section by immunostaining with anti-*B*. *anthracis* serum co-localized with the CD11b+ cells consistent with the expected phagocytic interaction of these cells with bacteria (S3 Fig).

**Fig 4:**
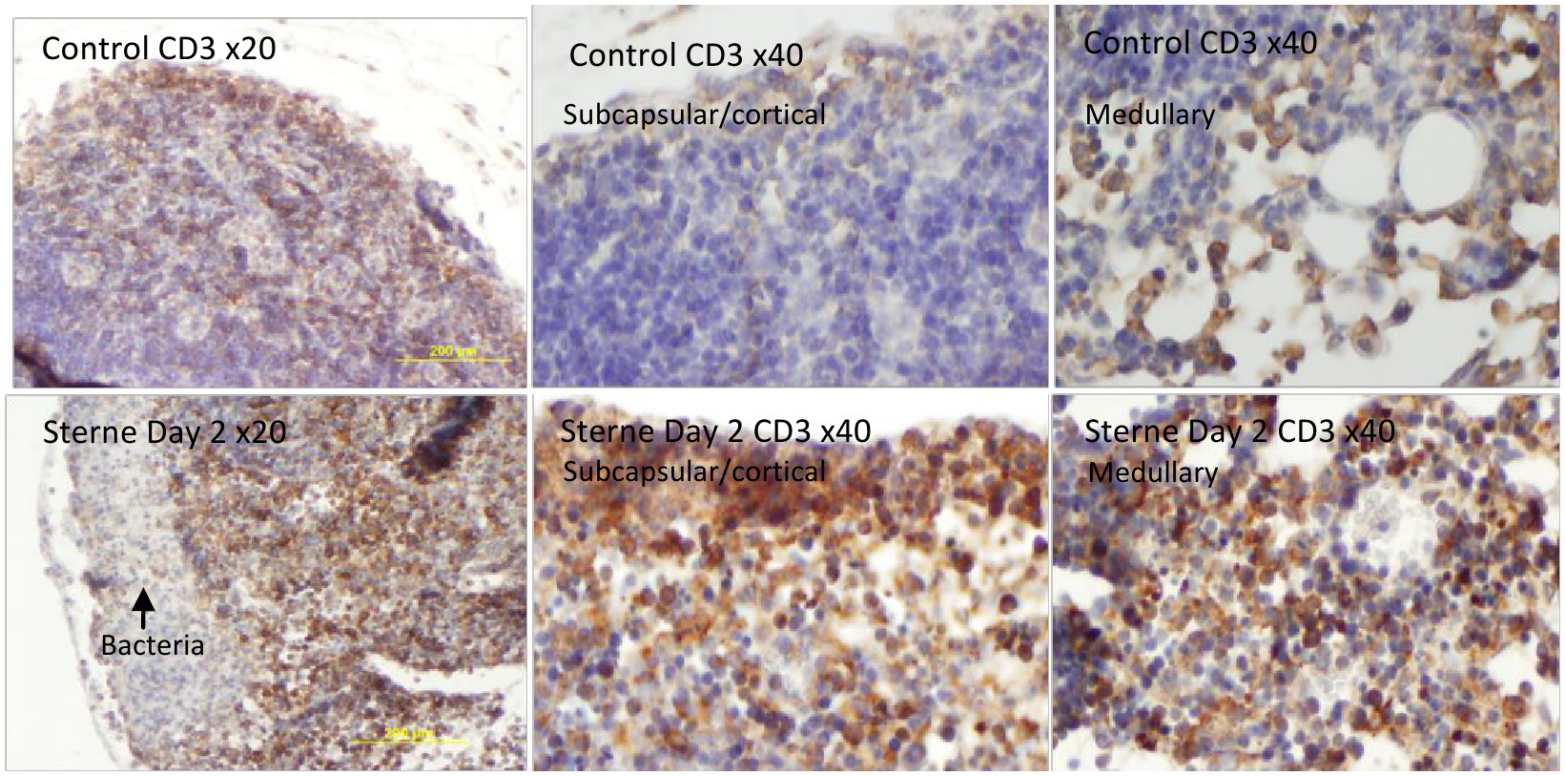
Immunohistochemical analysis of CD3 in formalin-fixed sections of popliteal LNs of naïve (top row) and *B*. *anthracis*-challenged mice (bottom row). Influx of CD3+ cells (stained brown) is visible in the subcapsular, cortical, and medullary regions of LNs at day 2 post infection with Sterne spores. A zone of bacterial growth is indicated by arrow. Slides were counterstained with hematoxylin (blue).

The CD3 marker is highly specific to T-lymphocytes different numbers of which were clearly visible in the cortical as well as the subcapsular and medullary regions of the LNs in naïve mice (Fig 4A). The infection caused a strong increase of the CD3+ cells demonstrating their participation in the immune response to infection. However, zones occupied by bacteria were almost free from the T cells which might be associated with their damage by *B*. *anthracis* pathogenic factors.

**Fig 5:**
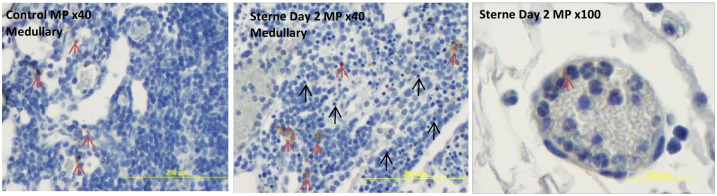
Immunohistochemical staining for neutrophil myeloperoxidase (MP) (brown) with hematoxylin counterstain (blue) in formalin-fixed sections of popliteal LNs of naïve and *B*. *anthracis* Sterne-challenged mice. Occasional MP-positive cells were detectable (red arrows) but the majority of infiltrating cells with PMN morphology were MP-negative. Many of the cells demonstrated pyknotic nuclei and were dead (black arrows). The migrating PMNs visible inside and outside of the blood vessels were mainly MP-negative (right panel).

To demonstrate an influx of neutrophils, the tissue sections were stained for a myeloperoxidase (MP) which is a marker of the bactericidal activity of mature neutrophils. Only occasionally the MP+ staining was detected in naïve and infected mice (Fig 5). Many of the infiltrating cells demonstrated pyknotic nuclei indicating their death by apoptosis. The migrating neutrophils were visible inside and outside of the blood vessels suggesting their appearance from the blood.

### RPMA of infected LNs reveals changes in the phosphoprotein levels

The LNs from infected and control mice were surgically removed from the euthanized mice and fixed in formalin for the subsequent preparation of paraffin blocks, which were sliced and mounted onto slides for the LCM. The slides were stained using standard hematoxylin technique for visual identification of the LNs under LCM microscope. The whole area of the LNs was cut out and transferred onto a polymer film for further lysis of the cellular content. For each LN, 30 slices were processed. The cell lysates were printed onto nitrocellulose membrane slides and probed with each of 65 different antibodies specific against phosphorylated forms of signaling proteins selected to monitor the molecular networks likely affected by bacterial exposure, namely survival, apoptosis, inflammation, growth, and immune response.

Of the tested signaling proteins, the levels of 60 proteins showed statistically significant (*p*<0.05) difference from those in the LN cells of control uninfected mice (Table 1). One of the most remarkable features of the LN responses was the impact of *B*. *anthracis* infection on the mitogen-activated signaling cascades and transcription. We detected a decreased phosphorylation of the pro-survival stress-activated protein kinase ERK1/2, p38, and SAPK/Jun amino-terminal kinases (JNK). The total levels of ERK1/2 and JNK also decreased, while the p38 showed some increase. However, the un-phosphorylated forms of these kinases were not expected to participate in signaling.

**Table 1:** Levels of phosphorylated signaling proteins and related proteins in LNs of *B*.*anthrasis* (Sterne)-infected mice relative to naïve mice.

Up-regulated*					Down-regulated*				
Protein	Day 1	Day 2	Day 3	Average per day	Protein	Day 1	Day 2	Day 3	Average per day
**Transcriptional response**									
Stat1	1.81	2.57	4.99	3.12	Stat6 (Y641)	0.86	0.87	0.54	0.76
Stat3 (Y705)	3.26	2.22	2.12	2.53	CREB (S133)	1.21	0.74	0.90	0.95
Stat5 (Y694)	1.47	1.61	1.54	1.54					
Stat1 (Y701)	1.21	1.46	1.73	1.47					
Stat6	1.51	1.04	1.55	1.37					
Stat3 (S727)	1.43	1.19	1.32	1.32					
IκB α	1.20	1.02	1.54	1.25					
NF-κB p65 (S536)	1.11	1.02	1.38	1.17					
ATF2 (T69/T71)	1.26	0.77	1.46	1.16					
Stat5	1.21	1.05	1.08	1.12					
**Mitogen-activated protein kinases**									
S6 (S235/S236)	1.61	1.49	3.77	2.29	ERK 1/2	0.43	0.35	0.23	0.33
S6 (S240/S244)	1.94	1.01	3.41	2.12	P38 MAPK (T180/Y182)	0.30	0.47	0.37	0.38
P38 MAPK	1.48	0.76	1.12	1.12	ERK1/2 (T202/Y204)	0.60	0.36	0.39	0.45
P70 S6 Kinase (T389)	1.09	1.04	1.18	1.10	SAPK/JNK	0.58	0.77	0.18	0.51
					SAPK/JNK (T183/Y185)	0.44	0.46	0.69	0.62
					P70 S6 Kinase(S371)	0.78	0.84	1.20	0.94
**Apoptosis, autophagy**									
Bim	3.82	4.93	5.73	4.83	HSP27(S82)	0.33	0.36	0.13	0.27
Survivin	1.52	2.01	4.37	2.63	FLIP	0.39	0.45	0.39	0.41
Puma	3.24	2.18	1.68	2.37	p53 (S15)	0.97	0.91	0.64	0.84
FADD (S191)	2.14	1.46	1.95	1.85	Bax	0.99	0.98	0.88	0.95
PARP cleaved (D214)	1.67	1.38	2.35	1.80	Bad	1.06	0.94	0.88	0.96
Caspase 3 cleaved (D175)	2.09	1.59	1.41	1.70					
XIAP	1.28	1.92	1.20	1.47					
Bcl-xL	1.25	1.27	1.24	1.26					
Caspase 9 cleaved (D330)	1.39	1.22	1.09	1.24					
Caspase 7 cleaved (D198)	1.30	1.39	0.91	1.20					
Beclin1	1.20	1.11	1.16	1.15					
LC3B	1.12	1.17	1.13	1.14					
Caspase 8	1.31	1.28	0.71	1.10					
**Lipid signaling/PI3K/AKT**									
SHIP1 (Y1020)	1.39	1.92	1.63	1.65	Akt (S473)	1.04	0.92	0.93	0.96
PTEN	1.71	1.22	1.45	1.46					
GSK 3α/β (S21/S9)	1.38	0.99	1.37	1.25					
AKT	1.44	0.90	1.34	1.23					
PTEN (S380)	1.40	0.94	1.26	1.20					
**Other protein kinases**									
Src (Y416) (+)	1.94	1.35	1.97	1.75	Lck (Y505) (-)	0.92	0.72	0.81	0.81
Src (Y517) (-)	1.94	1.35	1.98	1.75					
**Other**									
IL 10	1.18	4.62	0.98	2.26	Proteosome 20S	0.84	0.97	0.91	0.91
TLR9	3.18	1.67	1.40	2.08	Chk2 (S33/S35)	1.19	0.90	0.67	0.92
Myeloperoxidase	2.25	1.84	1.81	1.99	Sumo1	1.26	1.18	0.50	0.98
CD68	2.32	2.13	1.46	1.97					
eNOS (S1177)	1.49	1.40	1.29	1.39					
eNOS (S113)	1.48	1.00	1.30	1.26					
HSP90	1.00	1.04	1.37	1.14					
Sumo 2/3	0.88	0.75	1.70	1.11					

The MAPKs are regulated by upstream MAPKKs which are well-known specific targets of the LeTx proteolytic activity [18,19]. The inhibition ERK and p38 by LeTx leads to a reduction in pro-inflammatory responses and the induction of apoptosis in MΦs and endothelial cells [12,20]. Consistent with the inactivation of p38 is the reduced phosphorylation of the heat-shock protein Hsp27, which is a target of the p38 pathways [21]. The inhibition of JNK is expected to blunt the response to a variety of environmental stresses delivered to the JNK cascade by small GTPases of the Rho family, inflammatory cytokines, growth factors, and G-protein-coupled receptor agonists [22]. JNK translocates to the nucleus where it regulates activity of multiple transcription factors.

The increased phosphorylation of S6 ribosomal protein (at S235/S236, S240/S244) indicated activation of the P70 S6 kinase (at T389) by growth factors and mitogens leading to the subsequent phosphorylation of the S6 ribosomal protein as one of its targets. The activated S6 is expected to increase the translation of proteins involved in cell cycle progression as well as ribosomal proteins and elongation factors necessary for translation [23].

The activation of transcription factors was evident in the overall increased phosphorylation of STAT 1 (Y701), STAT 3 (Y705 and S727), STAT5 (Y694), NF-κB p65 (S536), and ATF2 (T69/T71). The total forms of STATs 1, 5 and 6 were also increased. However, the levels of several factors fluctuated or even decreased in the case of STAT 6 (Y641). This behavior likely reflected the presence of multiple competing stimuli controlling the transcription factor regulation. For example, CREB phosphorylation is expected to be sensitive to signals from several pathways including MEK1, 2, 5, and ERK3, in addition to the bacterial factors such as LeTx and EdTx [24,25].

Among the pro-apoptotic proteins there was an increase in caspase 3 cleaved (D175); caspase 7 cleaved (D198); PARP cleaved (D214); FADD (S194) adapter coupled to death signaling; caspase-8; and Puma promoting mitochondrial apoptosis through p53 pathway. A robust increase was also detected in the case of Bim antagonizing anti-apoptotic members of the Bcl-2 family, perhaps in response to the elevated levels of anti-apoptotic Bcl-xL and XIAP. Marginal responses (exceeding the statistical estimate of 95% reliability of *ca*. ±14% only at one time point) were detected for p53 (S15) activating apoptosis in response to myriad of stressors, Bax and Bad. No considerable autophagic activation was indicated by the levels of LC3B and Beclin1. The typical markers of apoptosis FLIP was found decreased by the infection which can be rationalized as a response aimed to increase the amount of activated caspase 8 by reducing the inhibiting effect of FLIP on its processing.

Surprisingly, the level of anti-apoptotic survivin was increased steadily up to more than 4-fold at day 3. This observation contrasted with the above results on the upregulation of caspases because this protein was expected to bind and inhibit caspase-3, controlling the checkpoint in the G2/M-phase of the cell cycle by inhibiting apoptosis and promoting cell division. It was also reported to inhibit mitochondrial apoptosis through caspase 9 [26]. Survivin is expressed highly in most human tumors and fetal tissue, but is normally reported absent in terminally differentiated cells [27]. Therefore, the upregulation of survivin may indicate the induction of the pro-survival signal which, reduced the pro-apoptotic effect of bacterial pathogenic factors, but was unable to overcome it.

Abrogation of the PI3K/AKT pro-survival signaling activation to the background level in response to the toxinogenic anthrax infection, as well as Rift Valley fever virus, was discovered by us previously in cell culture and circulating blood cells [25,28]. In agreement with these observations the phosphorylation of the global regulator of survival pathways serine/threonine kinase AKT at its major activating site S473 was not induced. Consistent with this, the levels of phosphorylation-activated phosphatases PTEN and SHIP1, the negative regulators of the PI3K/Akt signaling pathway, were increased. However, the phosphorylation of the downstream AKT target, the proapoptotic glycogen synthase kinase 3 (GSK-3) at the sites S21/S9 (for α/β isoforms, correspondingly), was increased. It suggests that in the absence of the AKT activity other pathways contributed the level of GSK-3 phosphorylation [29].

The non-receptor tyrosine protein kinase Src with a broad-spectrum activity promotes survival, angiogenesis, proliferation and invasion pathways [30]. Its phosphorylation at the activating and inactivating sites (Y416 and Y517, respectively) was almost equally increased. Judging by the elevated level of SHIP1 (Y1020) phosphatase which is a substrate of Src kinase, the latter was activated in the infected LNs. Another Src family tyrosine kinase Lck essential for T-lymphocyte activation and differentiation showed a decreased phosphorylation at the inhibiting site Y505. In line with the activation by Src was the increased phosphorylation of eNOS at the stimulating site Ser1177 in response to oxidative stress [31] implicated in anthrax pathology [32]. However, the inhibiting site Ser113 [33] also displayed an increased phosphorylation thus precluding a conclusion on the functional status of this enzyme.

The protective responses of the host were evident in the up-regulation of innate receptor TLR-9. The increased level of neutrophil myeloperoxidase supported our histopathological assessment of neutrophils transmigration to LNs from blood (Fig 5). The heat-shock protein HSP-90 was increased only at day 3 with no discernable effect on its target the 20S proteasome subunit. The response to the DNA damage during infection through sensing checkpoint kinase Chk2 was decreased. Changes in the levels of the small ubiquitin-related modifiers Sumo 1/2/3 indicated a pathogen-mediated interference with sumoylation of host proteins. This type of post-translational modifications similar to ubiquitination may represent a novel anthrax pathogenic mechanism potentially relevant to stress response and other biological processes [34].

The RPMA analysis demonstrated an increased level of CD68 (macrocialin) [35]. This transmembrane glycoprotein is highly expressed by human monocytes and tissue MΦs where it can be found in lysosomes and endosomes with a smaller fraction circulating to the cell surface. It binds to tissue- and organ-specific lectins or selectins. The protein is also a member of the scavenger receptor family clearing cellular debris, promoting phagocytosis, and mediating the recruitment and activation of MΦs. Macrosialin was not previously reported in association with anthrax infection.

### Immunohistochemical analysis of ERK, Puma, survivin, and macrosialin

**Fig 6:**
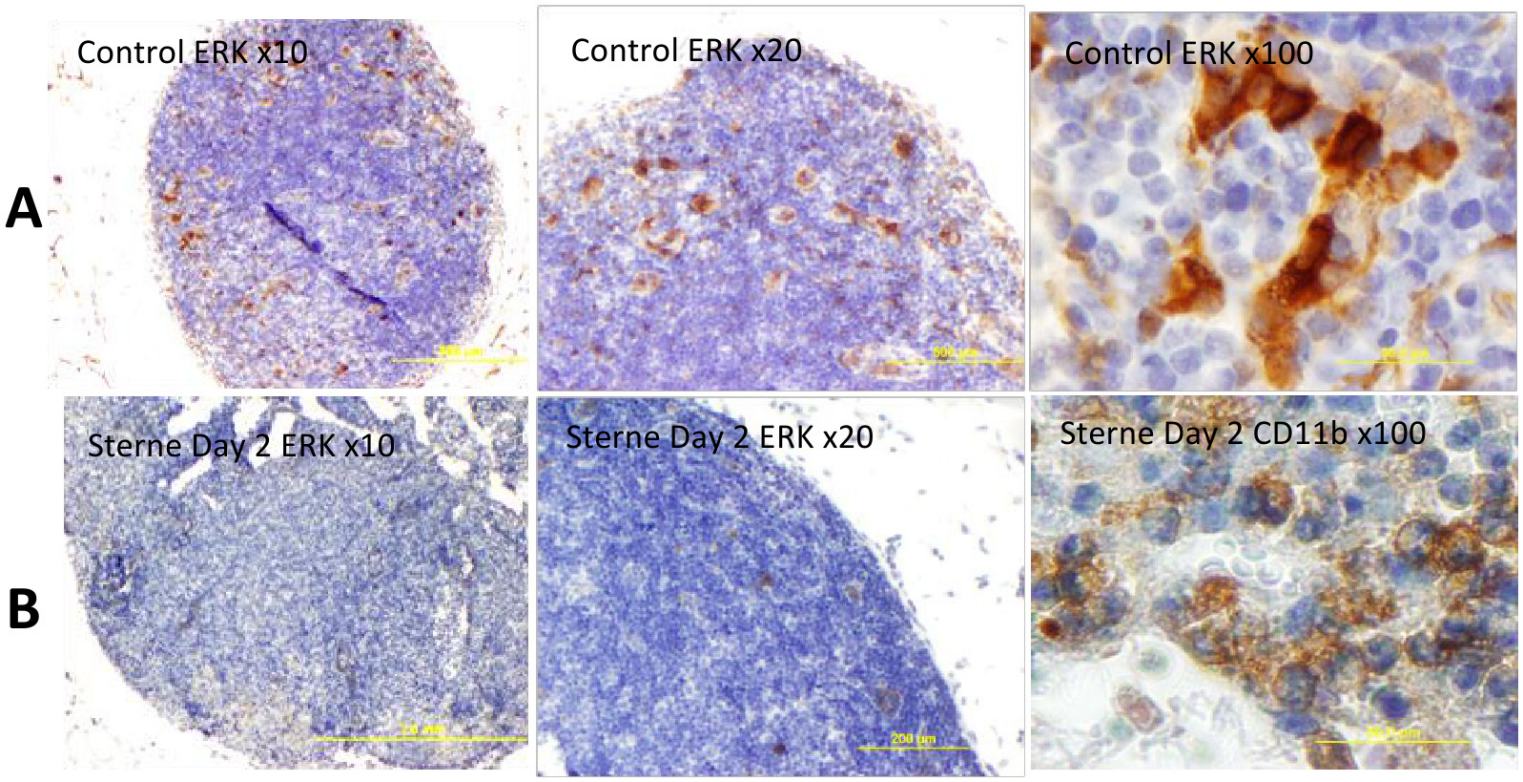
Immunohistochemical staining of formalin-fixed sections of popliteal LNs for phosphorylated ERK1/2 (brown) in naïve (A) and *B*. *anthracis* Sterne-challenged mice (B) with hematoxylin counterstain (blue) at different magnifications. The morphology of a typical CD11b staining is shown for comparison with the ERK1/2 one (right panels).

**Fig 7:**
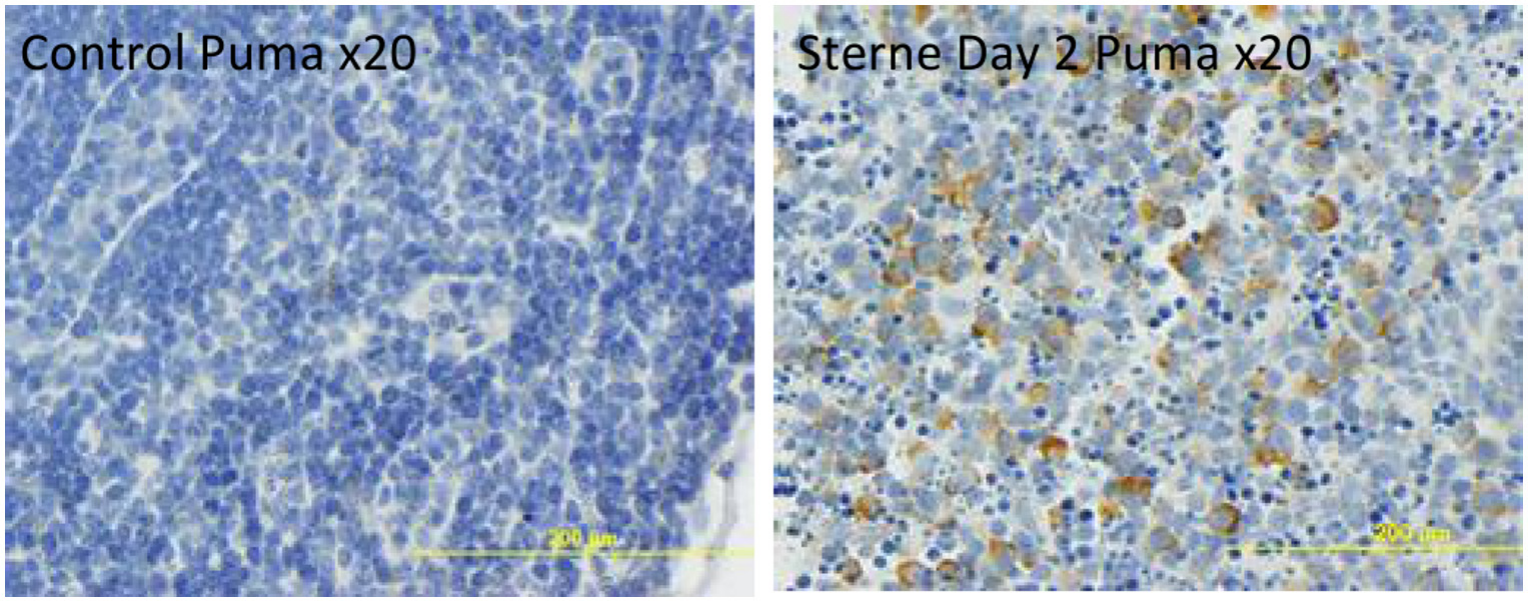
Immunohistochemical staining of formalin-fixed sections of popliteal LNs for Puma (brown) in control (left panel) and *B*. *anthracis* Sterne-challenged (right panel) mice. **Hematoxylin counterstain (blue) was used.** The dSterne-challenged mice demonstrated to Puma stain (not shown).

To substantiate the RPMA findings presented above we carried out the immunohistochemical analysis of LN tissue sections for selected proteins. The ERK+ cells were clearly visible in LNs of naïve mice and showed a characteristic morphology of the DC11b+ MΦs/DCs (Fig 6A). In agreement with the RPMA results, the staining was strongly reduced in the LNs from infected animals (Fig 6B). The bulk of the tissue did not stain for ERK indicating that neither T nor B cells were involved in the ERK response. However, the increased level of Puma which is pro-apoptotic for activated T-and B-cells [36–38] suggested that in pre-mortal animals the lymphocytes in the infected LNs were in the process of apoptosis. Fig 7 confirmed the up-regulation of Puma and demonstrated a staining pattern in the cortical region consistent with the response of CD3+ T-cells.

**Fig 8:**
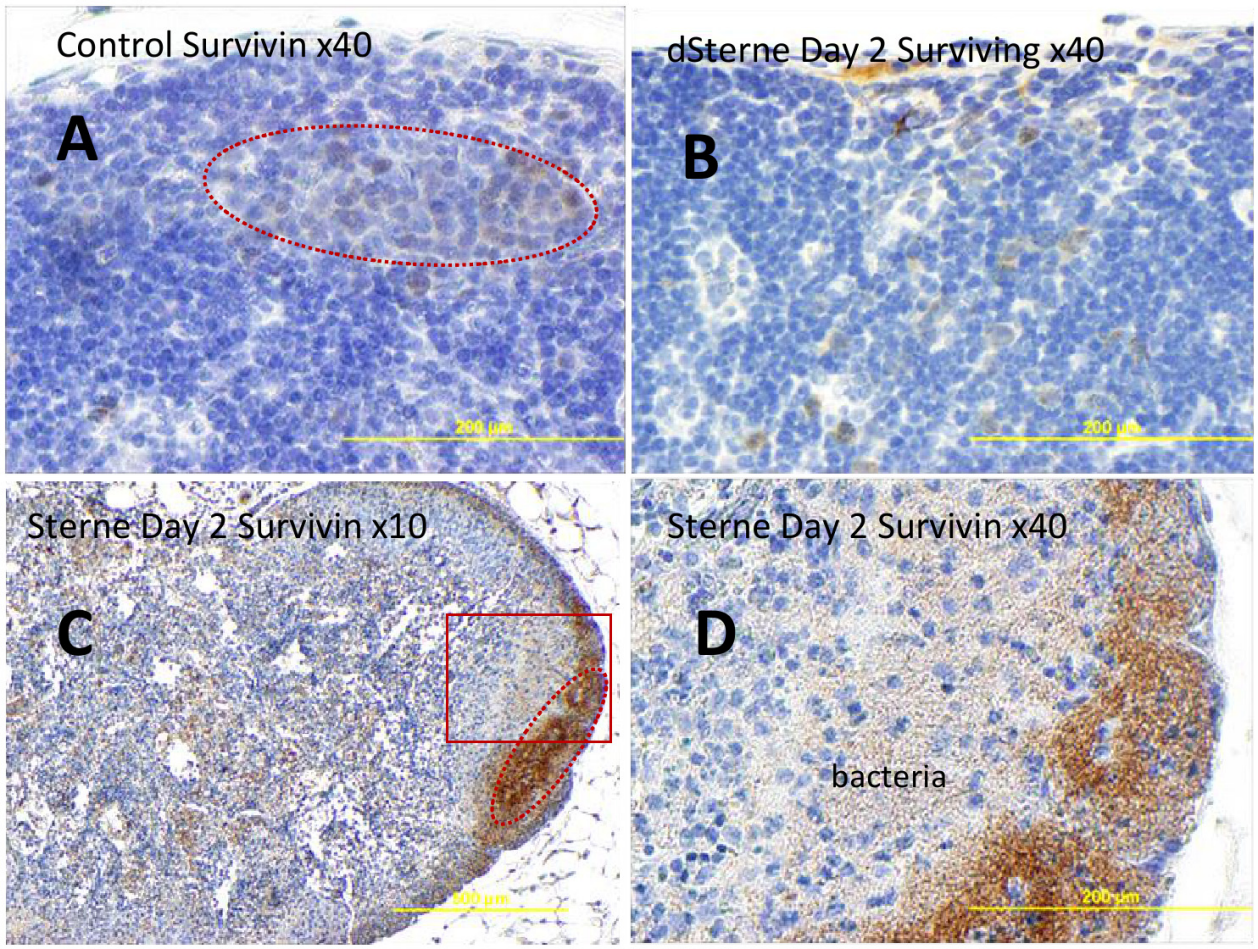
Immunohistochemical staining of formalin-fixed sections of popliteal LNs for survivin (brown) in naïve (A) and *B*. *anthracis* Sterne-challenged mice (B-D) at different magnifications. Hematoxylin counterstain (blue) was used. The majority of survinin+ cells (immunostained brown) localize to the cortical zones morphologically similar to B-cell lymphoid follicles (dotted lines in A and C) overlapping with bacteria in infected mice (bottom panels C, D). The boxed region in the left bottom panel C is magnified on the right.

The increased expression of survivin in the infected LNs was unexpected because generally it is not observed in adult tissues [27]. However, a low background level of survivin staining was detectable in the uninfected LNs. The Sterne-infected mice showed an increased amount of survivin detectable all over the LN tissue, especially intense in the cortical areas overlapping with the zones of bacterial proliferation (Fig 8).

**Fig 9:**
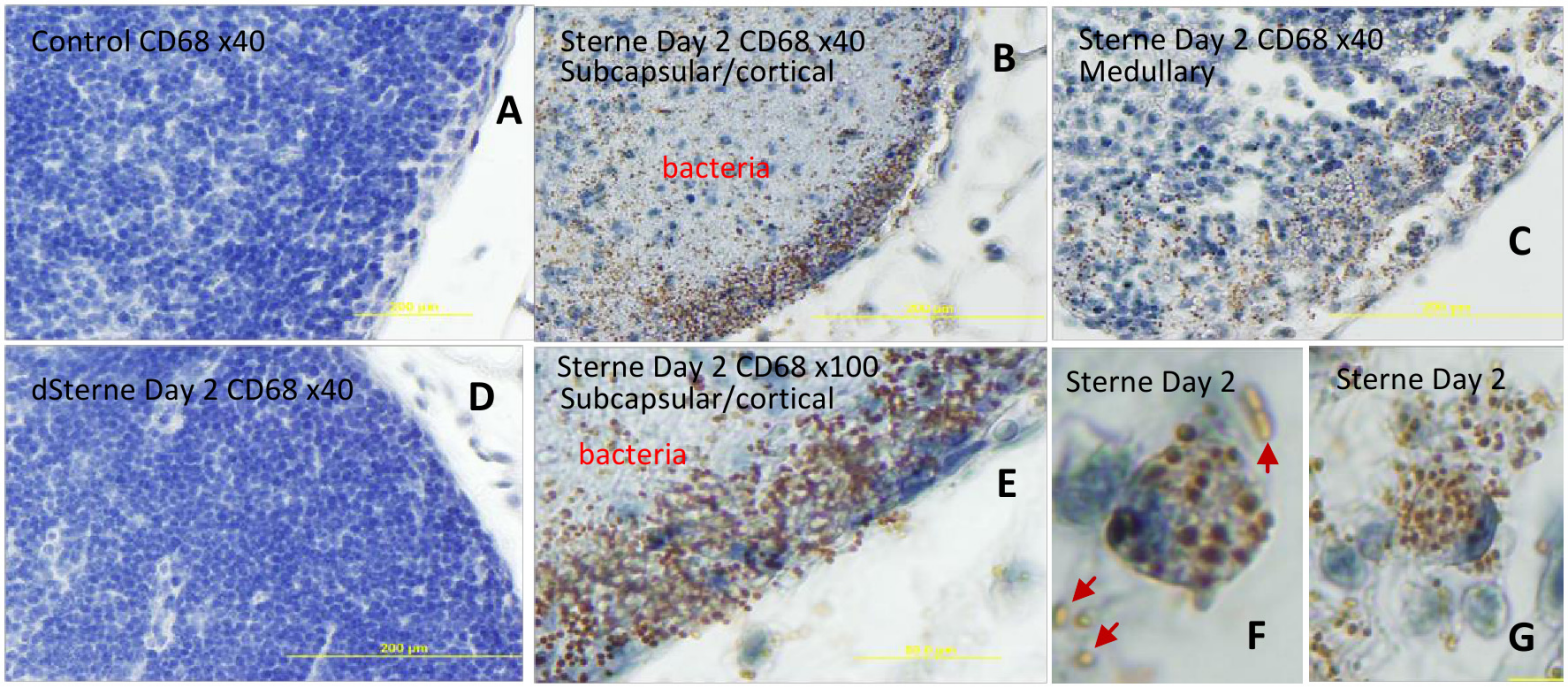
Immunohistochemical staining of formalin-fixed sections of popliteal LNs for macrosialin/CD68 (brown) in naïve (A) and *B*. *anthracis* Sterne-challenged mice (B) with hematoxylin counterstain (blue) at different magnifications. CD68 expression is induced in the Sterne-infected LNs (B,C,E). The staining demonstrates a punctate pattern mainly in subcapsular (B) and medullary (C) regions overlapping with bacteria (B, E). Control uninfected LNs and dSterne-infected LNs stain negative for CD68 (A, D, respectively). Phagocytic cells contain ingested bacteria with CD68 “caps” (F,G). The phagocytic uptake seems to be required for the staining of bacteria because the un-phagocytosed cells do not demonstrate the staining (F, arrows).

Immunostaining of macrosialin/CD68 typically expressed by monocytes/MΦs [39] revealed a punctate pattern in the areas occupied by bacteria (Fig 9). CD68 plays a role in phagocytic activities of tissue MΦs, both in intracellular lysosomal metabolism and extracellular cell-cell and cell-pathogen interactions [40]. CD68 rapidly recirculates from endosomes, lysosomes to the plasma membrane [35,39]. Closer examination showed that the CD68 protein was associated with the polar regions of the bacterial chains (Fig 9E–9G). The phagocytic cells (presumably MΦs) observed in the LNs contained a large number of bacteria with CD68 caps. Fig 9F and 9G shows such a MΦ in the process of releasing the intracellular bacteria containing the CD68 caps. However, the non-phagocytosed bacteria next to the MΦ appear to be unstained (Fig 9F, arrows) suggesting that phagocytosis was required for the staining.

## Discussion

We previously used the RPMA to characterize the interaction of cultured lung epithelial cells with secreted pathogenic factors of *B*. *anthracis* using cell lysates and to analyze signaling proteins in the intra-nodal lymph of spore-challenged mice [41]. In this study we for the first time applied the RPMA technique for the analyses of signaling proteins in the LN tissue to capture the phosphoprotein signaling *in situ*. We used the intradermal/subcutaneous challenge model in which the infectious spores of the toxinogenic Sterne strain were injected into the foot pads of mice for a direct delivery into the draining popliteal LNs. Although this and a similar model of cutaneous ear infection [42,43] are useful to characterize the progression of the disease in the lymphatics, which is believed to be one of the earliest targets of anthrax infection [1,44], the information on it available in the literature is rather limited.

One of the important LN functions is the filtration of microbes from the lymph by the reticular meshwork traversing the cortical and medullary sinuses. This process is aided by the phagocytic uptake and inactivation of infectious agents by macrophages (MΦs) and reticular cells. We found that the injected spores quickly disseminated from the site of challenge to the draining LNs and further to the bloodstream. The un-germinated, heat-resistant spores as well as the vegetative bacteria were detected in the spleen as early as 3 h post challenge (the earliest time point tested). This finding demonstrated that the systemic dissemination of the spores in our model did not require the onset of the hemorrhagic lymphadenitis as it’s currently accepted based on the autopsies of anthrax patients and experimental animals [1,4]. While the extensive hemorrhages were obvious in the pre-mortal animals, we detected no tissue damage at the 3-h time point (not shown).

Our results indicated that the filtration capacity of the LNs and the bactericidal activity of the innate immune response (including the activity of the phagocytic cells such as MΦs) were insufficient to limit the spread of the infectious material and ultimately eliminate infection. This conclusion is consistent with the observations that the lymphatic system remains rather permeable to the injected spores (which therefore may migrate all the way through the lymphatics and reach the bloodstream at the thoracic duct). The hydraulic radius of the sinus is likely very large and allows not only soluble molecules but also particles and cells to float through the meshwork of resident cells [16], until the infectious process triggers the inflammatory response leading to the increased retention of bacteria in medullary LN sinuses [15]. However, it has to be taken into account that much lower doses of administered spores will likely be within the capacity of the host to stop the progression of disease. This consideration explains the results of Weiner *et al*. [43] who reported that after the debridement of the inoculation site as a major site of infection the early delivery of spores into LNs (at the level of thousand PFUs) is not sufficient to establish a disseminated infection. In our experiments, the site of injection continued to supply spores to the LNs and spleen for at least 24 h.

In addition to the bacterial dissemination discussed above, another remarkable feature of the infection in our model was a high level of bacterial proliferation in the LNs. Bacteria occupied large areas of LNs demonstrating massive death of CD11b+ MΦs and CD3+ lymphocytes. The intense karyolysis (disappearance of the nuclear staining) indicated the onset of the tissue necrosis following cell death. The neutrophils infiltrating the LN from blood vessels in response to infection demonstrated low bactericidal capacity judged by the low frequency of MP+ cells, consistent with the incapacitation of neutrophils by anthrax toxins [24,45–47]. We conclude that the pre-mortal animals (tested at day 2 post challenge) could not control the virulent Sterne infection in the LNs which overwhelmed the protective capacity of immune system. In agreement with this, our previous proteomic analysis of the intra-nodal lymph during Sterne infection in the same animal model revealed a broad-spectrum shutdown of main cellular functions belonging to glycolysis, citrate cycle, metabolism of pyruvate, fatty acids, amino acids, and purines [41]. In contrast to our data the authors of [43] reported no heat-sensitive bacteria even at 72 h post injection but did not support their observation by histopathological analysis. This discrepancy might reflect bacterial lysis by distilled water used in [43] during the LN homogenization procedure.

The LNs are traditionally difficult to analyze, especially in rodent models due to the limited amount of material in the LN samples and sensitivity of the analytical procedures. These limitations are especially important in the host response signaling studies requiring analysis of a large variety of proteins in a small number of cells obtained from a local tissue microenvironment. In this report we demonstrated an experimental procedure combining the power of RPMA and LCM which allowed a reliable quantitative detection of a small amount of signaling phosphoproteins from the formaldehyde-fixed tissue slices. The phosphoproteins bear a wealth of information on the health status of the tissues and have a potential to be used as sensitive biomarkers. However, limited data are available regarding their role in LNs during anthrax infection. The specific antibodies we used in the RPMA assay served a double purpose of quantifying the material extracted from LNs and identifying the cells responding with the particular antigen after the immunostaining of the tissue.

In our characterization of the LN signaling we chose to detect the proteins belonging to diverse groups representing critical cellular signal transduction pathways. Among those the mitogen-activated stress response through MEK kinases is the established target of the proteolytic activity of the anthrax LeTx [24]. MΦs and DCs typically express the CD11b marker implicated in various adhesive interactions of monocytes, MΦs and granulocytes as well as in mediating the uptake of complement coated particles. MΦs and DCs have long been implicated as the cell types uniquely sensitive to the LeTx [24,44]. As seen in Fig 6, the ERK+ cells in control and infected mice represented a small fraction of LN cells and demonstrated the staining pattern similar to that of CD11b+ cells. While the infectious process reduced the ERK response, the amount of CD11b increased substantially (Fig 3). The majority of the staining was associated with a large amount of cellular debris consistent with the massive influx of phagocytes and their destruction by bacteria at the sites of infection, which may take place through different mechanisms. One possibility consists in the direct lysis of MΦs by the secreted bacterial phospholipases, the pore-forming toxin anthrolysin O, or the LeTx [44,48]. Alternatively, these pathogenic factors could also be responsible for the “inside-out” MΦ lysis by the intracellular spores or vegetative bacteria after their engulfment by the MΦs. This suggestion is consistent with our novel finding demonstrating the interaction of CD68/macrosialin with *B*. *anthracis* vegetative bacteria.

CD68 is a heavily glycosylated transmembrane protein of 87–115 kDa which is restricted mainly to cells of monocyte—macrophage lineage) [39]. This protein participates in the phagocytic uptake of microbial antigens, their intracellular delivery to the phagolysosome and antigen presentation. In MΦs, macrosialin is mainly localized in lysosomes and endosomes and rapidly exchanges with a smaller subtraction of macrosialin on the cell surface [35]. Exchange with intracellular pools occurs at an extremely high rate. The exact function of macrosialin is not known. It has been described as a member of the lysosomal/endosomal associated membrane glycoprotein (LAMP) family [39] and a member of the scavenger receptor family which recognizes a wide range of anionic macromolecules including oxidized low density lipoprotein, damaged apoptotic cells and surface antigens of microorganisms [35,40]. Information on CD68/macrosialin role in microbial infections is almost inexistent, although it was shown as the receptor on the macrophage surface for intercellular adhesive molecule from *Leishmania* species (ICAM-L) [49]. We present the first observation of CD68 participation in anthrax infection.

We suggest that the presence of CD68-stained bacteria in the Sterne-infected LNs reflects their prior direct interaction with the MΦs, perhaps during the germination of engulfed spores followed by intracellular multiplication of vegetative bacteria or after engagement of the pathogen with the CD68 exposed on the MΦ surface. An alternative mechanism when CD68 (or its fragment) interacted with bacteria after its release from MΦs into the LN environment seems unlikely because CD68 is a transmembrane protein. Supporting this conclusion, the amount of CD68 associated with bacteria was higher in the subcapsular regions where the majority of MΦs were expected to reside, in comparison with the deeper cortical areas (Fig 9B and 9E). It also explains a large amount of CD11b+ debris in the infected LNs (Fig 3) presumably reflecting a massive influx of MΦs and their consequent lysis after ingestion of the spores. It remains to be determined if the association of CD68 with bacteria took place post-delivery of the spores to the LNs in agreement with the jailbreak model or on the way of the spores to the LNs within the migrating MΦs according to the Trojan horse model. In any case, our data unequivocally demonstrate the elimination of MΦs in the LNs as a part of the pathogen’s immunosuppressive strategy.

One of the most remarkable features of the LN infection by the Sterne strain is the upregulation of transcriptional factors STATs (especially strong for phosphorylated STAT3). To our knowledge, this topic is novel to anthrax research. It was first noticed in our previous analysis of the soluble phosphoproteome [41]. The STAT family transcriptional factors participate in the JAK-STAT pleiotropic cascades allowing the cells to transduce signals for a large number of hormones, growth factors, and cytokines. STAT1 and STAT3 genes are specifically activated by phosphorylated forms of these proteins, respectively, resulting in large and prolonged increases in the levels of unphosphorylated STATs [50].

Depending upon the particular stimulus or cell type, STATs can mediate either pro-apoptotic or anti-apoptotic signals. STAT1 is important for transducing pro-apoptotic signals whereas STAT3 and STAT5 have been implicated in promoting cell survival. The pathogenic effects of STATs have been reported for some viral infections and cancer [51–54]. The bacterial periodontal pathogen *Porphyromonas gingivalis* invasion transiently inhibited *Pseudomonas aeruginosa*-induced apoptosis in respiratory epithelial cells *via* the STAT3 signaling pathway [55,56]. The activated STAT3 up-regulated the downstream anti-apoptotic survivin and Bcl-2 while down-regulating the pro-apoptotic death promoter Bad and caspase-3, thus aiding the survival of *P*. *gingivalis* within epithelial cells.

Consistent with the activation of STATs, in our experiments the apoptosis-related changes during infection were also accompanied by the up-regulation of survivin which plays a physiologically important role in response to tissue damage and homeostatic imbalance. Its expression is generally limited to pathological states, such as cancer, and is not observed in adult tissues [26]. Although recent data suggest roles for survivin in normal cells including T-cells, hematopoietic progenitor cells, vascular endothelial cells, liver cells, gastrointestinal tract mucosa, erythroid cells, and polymorphonuclear cells, survivin expression is significantly higher in transformed cells suggesting a pathological role for the protein [27].

Reports on the role of survivin in the infectious disease are limited. In addition to the mentioned above *P*. *gingivalis* infection, *Varicella-zoster* virus induces survivin to promote replication and skin pathogenesis. Survivin was also found induced in response to the GSK3β inhibitor during Venezuelan equine encephalomyelitis infection in astrocytes [57]. *H*. *pylori* infection leads to loss of survivin, presumably as a consequence of the changes in interactions with chaperones, such as Hsp90, which is essential for the stability of survivin [58]. The pro-survival activities of STATs and survivin in our experiments may reflect the response of the pathogen or the host aimed to delay the onset of apoptosis. The pathogenic consequence of such a delay requires further analysis.

In summary, this study presents the first description of the murine footpad *B*. *anthracis* challenge model and LN phosphoprotein responses associated with anthrax infection. We analyzed changes in the levels of 65 proteins many of which are directly relevant to the immune response, cell death and survival. Our RPMA analysis revealed a complex picture of the host cell signaling in response to the pathogenic insult. The remarkable feature of the host-pathogen interaction was the presence of signaling tendencies opposing each other, such as the concomitant activation of the pro- and anti-apoptotic proteins, emphasizing the notion [27] that the net biological outcome of the disease is determined by the relative impact of the microbial insult and the protective capacity of the host. The responses we detected reflect cumulative signals from different cells types and locations within the complex structure of the LN tissue. It may result in the “dilution” of the signal by the non-responders if the latter are present at high numbers. In the future studies we plan to address the spatial distribution and frequencies of the responding cells in order to delineate their relative contributions.

The experimental technique we used in this report for the analysis of cell signaling *in situ* after challenge with the attenuated Sterne strain involves inactivation of the infectious material by the formaldehyde. It offers a possibility to carry out comparative signaling studies with fully virulent strains at the biological safety level 2 (BSL-2) using tissue sections prepared in BSL-3 experiments. Such analysis is much needed in the field to avoid a potential controversy associated with the use of different strains in experiments addressing the contribution of pathogenic factors to *B*. *anthracis* virulence.

## Supporting Information

S1 Fig Fluorescent pNIPAm NPs labelled with Alexa Fluor 555 (yellow) quickly migrate to sub-capsular (white arrows) and medullar regions of popliteal LNs of mice (red arrows) after injection of suspension (20 μl in PBS) into mouse hind foot pads for 30 min. The popliteal LNs were surgically removed for histologic evaluation. The LNs were paraffin-embedded after fixation with paraformaldehyde, and the 5 μm tissue slices were mounted onto glass slide. The particles were observed at 555/570 nm using Olympus BX51 microscope with a TRITC filter set.
(TIF)

S2 Fig Immunohistochemical analysis of CD11b in formalin-fixed sections of popliteal LNs from *B*. *anthracis*-challenged mice. Macrophages but not neutophils are highly CD11b-positive. Macrophages (brown stain) are black arrows, neuthrophils (blue stain, fragmented nuclei) are red arrows. Bacteria (no stain) are shown by green arrows. Numerous neutrophils with condensed nuclei are also present.
(TIF)

S3 Fig The CD11b+ cells co-localize with bacteria in dSterne-infected LNs. Two consecutive slices of formalin-fixed LNs from dSterne-infected mice were immunostained for CD11b (left panels) and B. anthracis (middle panels). The images of two different areas from each section (top and bottom rows) were taken and electronically separated into layers of RBG colors. The layers corresponding to the diaminobenzidine stain were assigned the green and red colors for CD11b and bacteria, correspondingly. The right panels demonstrate the overlays where the overlapping green and red colors indicate a co-localization of CD11b+ cells with bacteria (arrows).
(TIF)

## References

[1] Inglesby T V, O’Toole T, Henderson DA, Bartlett JG, Ascher MS, Eitzen E, et al Anthrax as a biological weapon, 2002: updated recommendations for management. JAMA. 2002 5 1;287(17):2236–52. 11980524 10.1001/jama.287.17.2236

[2] Beierlein JM, Anderson AC. New developments in vaccines, inhibitors of anthrax toxins, and antibiotic therapeutics for Bacillus anthracis. Curr Med Chem. 2011 1;18(33):5083–94. 22050756 10.2174/092986711797636036 PMC3644987

[3] Glomski IJ, Piris-Gimenez A, Huerre M, Mock M, Goossens PL. Primary involvement of pharynx and peyer’s patch in inhalational and intestinal anthrax. PLoS Pathog. 2007 6;3(6):e76 17542645 10.1371/journal.ppat.0030076 PMC1885272

[4] Weiner ZP, Glomski IJ. Updating perspectives on the initiation of Bacillus anthracis growth and dissemination through its host. Infect Immun. 2012 5;80(5):1626–33. 10.1128/IAI.06061-11 22354031 PMC3347428

[5] Mina B, Dym JP, Kuepper F, Tso R, Arrastia C, Kaplounova I, et al Fatal inhalational anthrax with unknown source of exposure in a 61-year-old woman in New York City. JAMA. 2002 2 20;287(7):858–62. 11851577 10.1001/jama.287.7.858

[6] Wulfkuhle JD, Edmiston KH, Liotta LA, Petricoin EF. Technology insight: pharmacoproteomics for cancer—promises of patient-tailored medicine using protein microarrays. Nat Clin Pract Oncol. 2006 5;3(5):256–68. 16683004 10.1038/ncponc0485

[7] Pierobon M, Wulfkuhle J, Liotta L, Petricoin E. Application of molecular technologies for phosphoproteomic analysis of clinical samples. Oncogene. 2014 3 10; 10.1038/onc.2014.16 24608425

[8] Espina V, Wulfkuhle JD, Calvert VS, VanMeter A, Zhou W, Coukos G, et al Laser-capture microdissection. Nat Protoc. 2006 1;1(2):586–603. 17406286 10.1038/nprot.2006.85

[9] Longo C, Patanarut A, George T, Bishop B, Zhou W, Fredolini C, et al Core-shell hydrogel particles harvest, concentrate and preserve labile low abundance biomarkers. PLoS One. 2009;4(3). 10.1371/journal.pone.0004763 PMC2651577 19274087

[10] Luchini A, Geho DH, Bishop B, Tran D, Xia C, Dufour R, et al Smart Hydrogel Particles: Biomarker Harvesting: One-step affinity purification, size exclusion, and protection against degradation. Nano Lett. 2008 1;8(1):350–61. 18076201 10.1021/nl072174l PMC2877922

[11] Luchini A, Fredolini C, Espina BH, Meani F, Reeder A, Rucker S, et al Nanoparticle technology: addressing the fundamental roadblocks to protein biomarker discovery. Curr Mol Med. 2010;10(2):133–41. 20196732 10.2174/156652410790963268 PMC2873152

[12] Popov SG, Villasmil R, Bernardi J, Grene E, Cardwell J, Wu A, et al Lethal toxin of Bacillus anthracis causes apoptosis of macrophages. Biochem Biophys Res Commun. 2002 4 26;293(1):349–55. 12054607 10.1016/S0006-291X(02)00227-9

[13] Spurrier B, Ramalingam S, Nishizuka S. Reverse-phase protein lysate microarrays for cell signaling analysis. Nat Protoc. 2008 1;3(11):1796–808. 10.1038/nprot.2008.179 18974738

[14] Long KM, Heise M. Safe and effective mouse footpad inoculation. Methods Mol Biol. 2013 1;1031:97–100. 10.1007/978-1-62703-481-4_12 23824892

[15] Smith RO, Wood WB. Cellular mechanisms of antibacterial defense in lymph nodes; pathogenesis of acute bacterial lymphadenitis. J Exp Med. 1949 12;90(6):555–66. 15394073 10.1084/jem.90.6.555 PMC2135929

[16] Lämmermann T, Sixt M. The microanatomy of T-cell responses. Immunol Rev. 2008 2;221:26–43. 10.1111/j.1600-065X.2008.00592.x 18275473

[17] Bachmann MF, Jennings GT. Vaccine delivery: a matter of size, geometry, kinetics and molecular patterns. Nat Rev Immunol. Nature Publishing Group, a division of Macmillan Publishers Limited. All Rights Reserved.; 2010 11;10(11):787–96. 10.1038/nri2868 20948547

[18] Duesbery NS, Vande Woude GF. Anthrax lethal factor causes proteolytic inactivation of mitogen-activated protein kinase kinase. J Appl Microbiol. 1999 8;87(2):289–93. 10475971 10.1046/j.1365-2672.1999.00892.x

[19] Vitale G, Pellizzari R, Recchi C, Napolitani G, Mock M, Montecucco C. Anthrax lethal factor cleaves the N-terminus of MAPKKS and induces tyrosine/threonine phosphorylation of MAPKS in cultured macrophages. J Appl Microbiol. 1999 8;87(2):288 10475970 10.1046/j.1365-2672.1999.00893.x

[20] Kirby JE. Anthrax lethal toxin induces human endothelial cell apoptosis. Infect Immun. 2004 1;72(1):430–9. 14688124 10.1128/IAI.72.1.430-439.2004 PMC343952

[21] Landry J, Lambert H, Zhou M, Lavoie JN, Hickey E, Weber LA, et al Human HSP27 is phosphorylated at serines 78 and 82 by heat shock and mitogen-activated kinases that recognize the same amino acid motif as S6 kinase II. J Biol Chem. 1992 1 15;267(2):794–803. 1730670

[22] Kyriakis JM, Avruch J. Mammalian mitogen-activated protein kinase signal transduction pathways activated by stress and inflammation. Physiol Rev. 2001 4;81(2):807–69. 11274345 10.1152/physrev.2001.81.2.807

[23] Magnuson B, Ekim B, Fingar DC. Regulation and function of ribosomal protein S6 kinase (S6K) within mTOR signalling networks. Biochem J. 2012 1 1;441(1):1–21. 10.1042/BJ20110892 22168436

[24] Guichard A, Nizet V, Bier E. New insights into the biological effects of anthrax toxins: linking cellular to organismal responses. Microbes Infect. 2012 2;14(2):97–118. 10.1016/j.micinf.2011.08.016 21930233 PMC3743078

[25] Popova T, Espina V, Bailey C, Liotta L, Petricoin E, Popov S. Anthrax infection inhibits the AKT signaling involved in the E-cadherin-mediated adhesion of lung epithelial cells. FEMS Immunol Med Microbiol. 2009 7;56(2):129–42. 10.1111/j.1574-695X.2009.00558.x 19416348 PMC2734923

[26] Altieri DC. Survivin—The inconvenient IAP. Semin Cell Dev Biol. 2015; 39:91–96. 10.1016/j.semcdb.2014.12.007 25591986 PMC4410054

[27] Mobahat M, Narendran A, Riabowol K. Survivin as a preferential target for cancer therapy. Int J Mol Sci. 2014 1;15(2):2494–516. 10.3390/ijms15022494 24531137 PMC3958864

[28] Popova TG, Turell MJ, Espina V, Kehn-Hall K, Kidd J, Narayanan A, et al Reverse-phase phosphoproteome analysis of signaling pathways induced by Rift valley fever virus in human small airway epithelial cells. PLoS One. 2010 1;5(11):e13805 10.1371/journal.pone.0013805 21072193 PMC2972203

[29] McCubrey JA, Steelman LS, Bertrand FE, Davis NM, Sokolosky M, Abrams SL, et al GSK-3 as potential target for therapeutic intervention in cancer. Oncotarget. 2014 5 30;5(10):2881–911. 24931005 10.18632/oncotarget.2037 PMC4102778

[30] Roskoski R. Src protein-tyrosine kinase structure, mechanism, and small molecule inhibitors. Pharmacol Res. 2015 2 3;94C:9–25. 10.1016/j.phrs.2015.01.003 25662515

[31] Thomas SR, Chen K, Keaney JF. Hydrogen peroxide activates endothelial nitric-oxide synthase through coordinated phosphorylation and dephosphorylation via a phosphoinositide 3-kinase-dependent signaling pathway. J Biol Chem. 2002 2 22;277(8):6017–24. 11744698 10.1074/jbc.M109107200

[32] Popova TG, Millis B, Chung M-C, Bailey C, Popov SG. Anthrolysin O and fermentation products mediate the toxicity of Bacillus anthracis to lung epithelial cells under microaerobic conditions. FEMS Immunol Med Microbiol. 2011 2;61(1):15–27. 10.1111/j.1574-695X.2010.00740.x 20946354 PMC3040846

[33] Shin E, Yeo E, Lim J, Chang YH, Park H, Shim E, et al Nitrooleate mediates nitric oxide synthase activation in endothelial cells. Lipids. 2014 5;49(5):457–66. 10.1007/s11745-014-3893-8 24664541

[34] Ribet D, Cossart P. SUMOylation and bacterial pathogens. Virulence. 1;1(6):532–4. 21178495 10.4161/viru.1.6.13449

[35] Kurushima H, Ramprasad M, Kondratenko N, Foster D, Quehenberger O, Steinberg D. Surface expression and rapid internalization of macrosialin (mouse CD68) on elicited mouse peritoneal macrophages. J Leukoc Biol. 2000 1 1;67(1):104–8. 10648004 10.1002/jlb.67.1.104

[36] Clybouw C, Fischer S, Auffredou MT, Hugues P, Alexia C, Bouillet P, et al Regulation of memory B-cell survival by the BH3-only protein Puma. Blood. 2011 10 13;118(15):4120–8. 10.1182/blood-2011-04-347096 21868573 PMC3204730

[37] Häcker G, Bauer A, Villunger A. Apoptosis in Activated T Cells—What Are the Triggers, and What the Signal Transducers? Cell Cycle. Taylor & Francis; 2014 10 28;5(21):2421–4. 10.4161/cc.5.21.3397 17102629

[38] Strasser A. The role of BH3-only proteins in the immune system. Nat Rev Immunol. 2005 3;5(3):189–200. 15719025 10.1038/nri1568

[39] Holness CL, da Silva RP, Fawcett J, Gordon S, Simmons DL. Macrosialin, a mouse macrophage-restricted glycoprotein, is a member of the lamp/lgp family. J Biol Chem. 1993 5 5;268(13):9661–6. 8486654

[40] Ramprasad MP, Terpstra V, Kondratenko N, Quehenberger O, Steinberg D. Cell surface expression of mouse macrosialin and human CD68 and their role as macrophage receptors for oxidized low density lipoprotein. Proc Natl Acad Sci U S A. 1996 12 10;93(25):14833–8. 8962141 10.1073/pnas.93.25.14833 PMC26222

[41] Popova TG, Espina V, Zhou W, Mueller C, Liotta L, Popov SG. Whole proteome analysis of mouse lymph nodes in cutaneous anthrax. PLoS One. 2014 1;9(10):e110873 10.1371/journal.pone.0110873 25329596 PMC4203832

[42] Corre J-P, Piris-Gimenez A, Moya-Nilges M, Jouvion G, Fouet A, Glomski IJ, et al In vivo germination of Bacillus anthracis spores during murine cutaneous infection. J Infect Dis. 2013 2 1;207(3):450–7. 10.1093/infdis/jis686 23148288

[43] Weiner ZP, Boyer AE, Gallegos-Candela M, Cardani AN, Barr JR, Glomski IJ. Debridement increases survival in a mouse model of subcutaneous anthrax. PLoS One. 2012 1;7(2):e30201 10.1371/journal.pone.0030201 22393351 PMC3290625

[44] Mock M, Fouet A. Anthrax. Annu Rev Microbiol. 2001 1;55:647–71. 11544370 10.1146/annurev.micro.55.1.647

[45] Garraud K, Cleret A, Mathieu J, Fiole D, Gauthier Y, Quesnel-Hellmann A, et al Differential Role of the Interleukin-17 Axis and Neutrophils in Resolution of Inhalational Anthrax. Infection and Immunity. 2012 p. 131–42. 10.1128/IAI.05988-11 22025514 PMC3255671

[46] Barua S, Iyer JK, Larabee JL, Raisley B, Hughes M a, Coggeshall KM, et al Toxin Inhibition of Antimicrobial Factors Induced by Bacillus anthracis Peptidoglycan in Human Blood. Infect Immun. 2013;81(10):3693–702. 10.1128/IAI.00709-13 23876807 PMC3811742

[47] Nguyen C, Feng C, Zhan M, Cross AS, Goldblum SE. Bacillus anthracis-derived edema toxin (ET) counter-regulates movement of neutrophils and macromolecules through the endothelial paracellular pathway. BMC Microbiol. 2012 1;12(1):2. 22230035 10.1186/1471-2180-12-2 PMC3277462

[48] Klichko VI, Miller J, Wu A, Popov SG, Alibek K. Anaerobic induction of Bacillus anthracis hemolytic activity. Biochem Biophys Res Commun. 2003 4 11;303(3):855–62. 12670489 10.1016/s0006-291x(03)00440-6

[49] Ng H-P, Chiang S-C, Chi Y, Lee S-T. Identification of macrosialin (CD68) on the surface of host macrophages as the receptor for the intercellular adhesive molecule (ICAM-L) of Leishmania amazonensis. Int J Parasitol. 2009 12;39(14):1539–50. 10.1016/j.ijpara.2009.06.001 19540239

[50] Battle TE, Frank DA. The role of STATs in apoptosis. Curr Mol Med. 2002 6;2(4):381–92. 12108949 10.2174/1566524023362456

[51] Cai Q, Verma SC, Choi J-Y, Ma M, Robertson ES. Kaposi’s sarcoma-associated herpesvirus inhibits interleukin-4-mediated STAT6 phosphorylation to regulate apoptosis and maintain latency. J Virol. 2010 11;84(21):11134–44. 10.1128/JVI.01293-10 20719954 PMC2953196

[52] Wang Z, Luo F, Li L, Yang L, Hu D, Ma X, et al STAT3 activation induced by Epstein-Barr virus latent membrane protein1 causes vascular endothelial growth factor expression and cellular invasiveness via JAK3 And ERK signaling. Eur J Cancer. 2010 11;46(16):2996–3006. 10.1016/j.ejca.2010.07.008 20709526

[53] Zhang Y, Zhang Y, Yun H, Lai R, Su M. Correlation of STAT1 with Apoptosis and Cell-Cycle Markers in Esophageal Squamous Cell Carcinoma. PLoS One. 2014 1;9(12):e113928 10.1371/journal.pone.0113928 25438156 PMC4250046

[54] Bok K, Prikhodko VG, Green KY, Sosnovtsev S V. Apoptosis in murine norovirus-infected RAW264.7 cells is associated with downregulation of survivin. J Virol. 2009 4;83(8):3647–56. 10.1128/JVI.02028-08 19211757 PMC2663291

[55] Li Q, Pan C, Teng D, Lin L, Kou Y, Haase EM, et al Porphyromonas gingivalis modulates Pseudomonas aeruginosa-induced apoptosis of respiratory epithelial cells through the STAT3 signaling pathway. Microbes Infect. 2014 1;16(1):17–27. 10.1016/j.micinf.2013.10.006 24140557

[56] Mao S, Park Y, Hasegawa Y, Tribble GD, James CE, Handfield M, et al Intrinsic apoptotic pathways of gingival epithelial cells modulated by Porphyromonas gingivalis. Cell Microbiol. 2007 8;9(8):1997–2007. 17419719 10.1111/j.1462-5822.2007.00931.x PMC2886729

[57] Kehn-Hall K, Narayanan A, Lundberg L, Sampey G, Pinkham C, Guendel I, et al Modulation of GSK-3β activity in Venezuelan equine encephalitis virus infection. PLoS One. 2012 1 4;7(4):e34761 10.1371/journal.pone.0034761 22496857 PMC3319612

[58] Valenzuela M, Bravo D, Canales J, Sanhueza C, Díaz N, Almarza O, et al Helicobacter pylori-induced loss of survivin and gastric cell viability is attributable to secreted bacterial gamma-glutamyl transpeptidase activity. J Infect Dis. 2013 10 1;208(7):1131–41. 10.1093/infdis/jit286 23847060

