# Supplementary figures and images for: Reverse-Phase Microarray Analysis Reveals Novel Targets in Lymph Nodes of Bacillus anthracis Spore-Challenged Mice
Source: PLoS One. 2015 Jun 19;10(6):e0129860. doi: 10.1371/journal.pone.0129860 (PMC4474663; doi:10.1371/journal.pone.0129860)

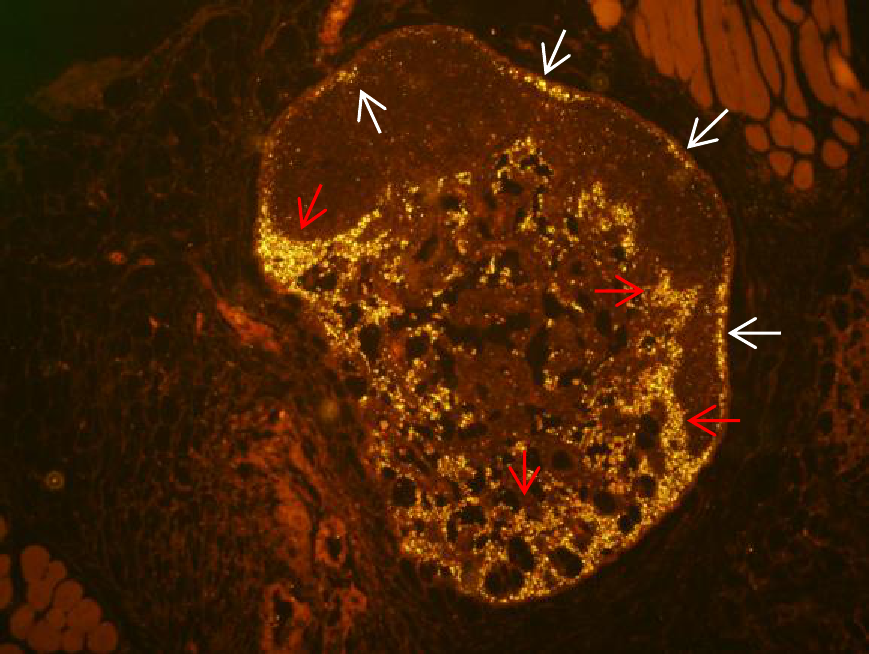

Supplement: S1 Fig — The popliteal LNs were surgically removed for histologic evaluation. The LNs were paraffin-embedded after fixation with paraformaldehyde, and the 5 μm tissue slices were mounted onto glass slide. The particles were observed at 555/570 nm using Olympus BX51 microscope with a TRITC filter set. (TIF) [file pone.0129860.s001.tif]

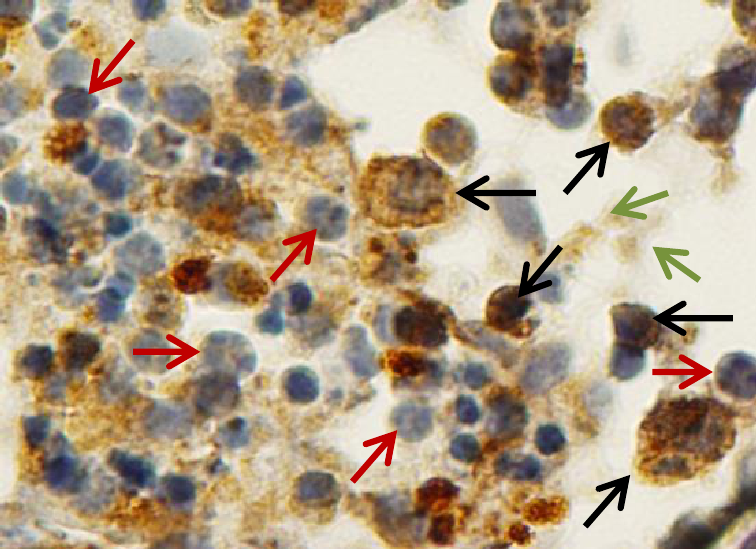

Supplement: S2 Fig — Macrophages but not neutophils are highly CD11b-positive. Macrophages (brown stain) are black arrows, neuthrophils (blue stain, fragmented nuclei) are red arrows. Bacteria (no stain) are shown by green arrows. Numerous neutrophils with condensed nuclei are also present. (TIF) [file pone.0129860.s002.tif]

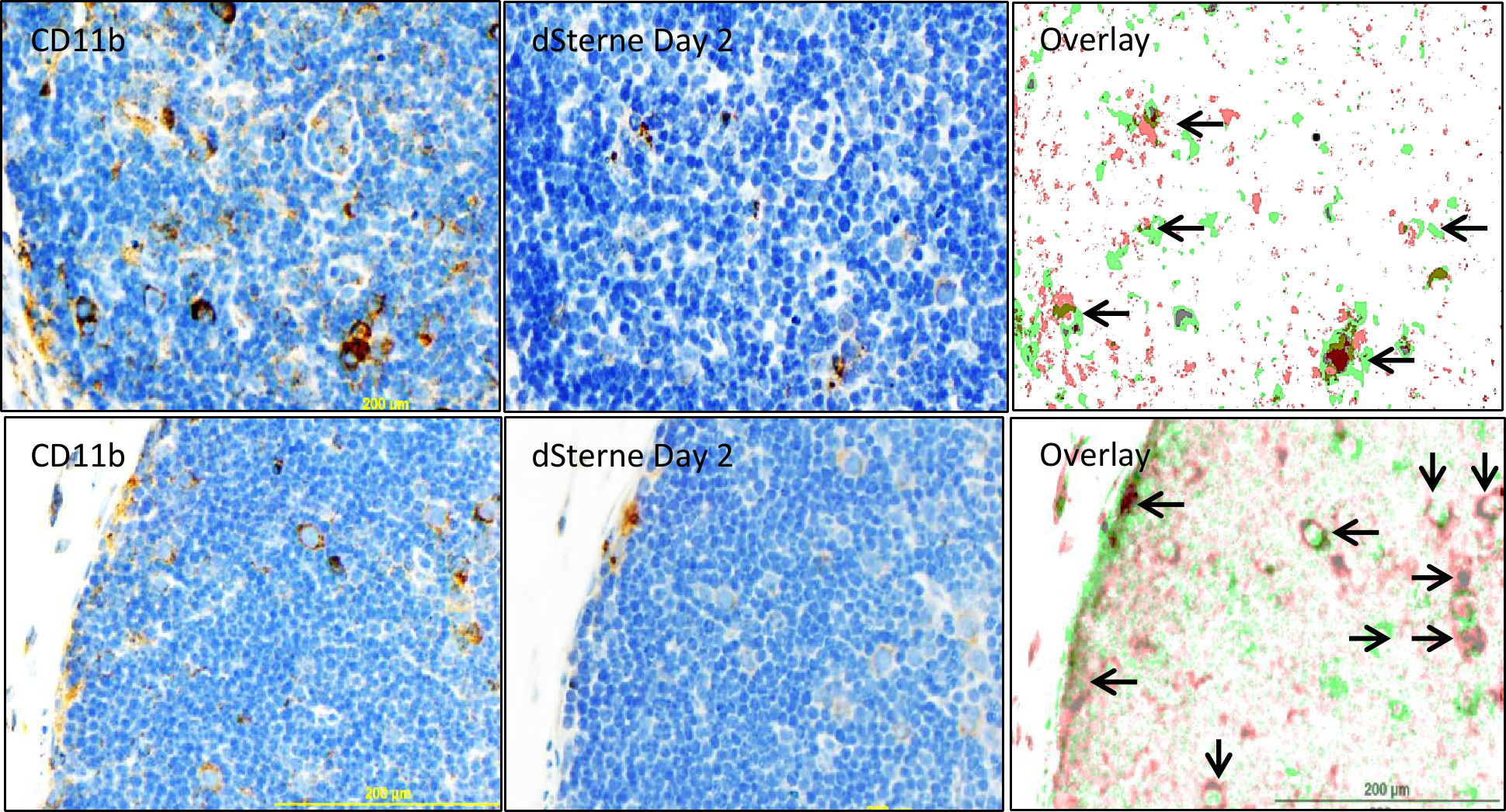

Supplement: S3 Fig — Two consecutive slices of formalin-fixed LNs from dSterne-infected mice were immunostained for CD11b (left panels) and B. anthracis (middle panels). The images of two different areas from each section (top and bottom rows) were taken and electronically separated into layers of RBG colors. The layers corresponding to the diaminobenzidine stain were assigned the green and red colors for CD11b and bacteria, correspondingly. The right panels demonstrate the overlays where the overlapping green and red colors indicate a co-localization of CD11b+ cells with bacteria (arrows). (TIF) [file pone.0129860.s003.tif]
